# Psychological heterogeneity and residential demands in early-stage aging: evidence from a human-environment systems framework

**DOI:** 10.3389/fpubh.2026.1808521

**Published:** 2026-07-13

**Authors:** Lin Yuan, Xiang Wang, Runze Liu, Zhaoyi Yang, Yan Zhang, Weicong Li

**Affiliations:** 1 School of Architecture and Art, North China University of Technology, Beijing, China; 2 College of Environment and Architectural Arts, Tianjin Academy of Fine Arts, Tianjin, China; 3 Faculty of Built Environment and Surveying, Universiti Teknologi Malaysia, Johor Bahru, Johor, Malaysia

**Keywords:** aging-friendly design, inclusive spatial design, psychological type, questionnaire clustering, residential demands

## Abstract

**Background:**

Research on home-based eldercare has increasingly moved beyond physical safety and accessibility to consider psychological wellbeing and everyday activity. However, older adults are still often treated as a homogeneous group. Less is known about how different psychological profiles are associated with residential preferences, activity patterns, and perceived environmental support among early-stage aging adults.

**Methods:**

This study focused on early-stage older adults and applied a human-environment systems framework to examine the relationships among psychological characteristics, residential conditions, and home-based activities. A total of 509 valid household questionnaires were collected in northern China between July and September 2025. Psychological profiles were identified using K-means clustering, and differences in residential preferences, activity patterns, and facility provision across psychological types were analyzed using descriptive statistics and inferential tests.

**Results:**

Three psychological types were identified. Overall, 45.2% of respondents preferred living adjacent to their children, while lower-income participants showed a stronger tendency toward co-residence. Shared family meals and household chores were the most frequent activities, whereas participation in fitness, hobbies, and shared entertainment was limited. The psychologically vulnerable group exhibited poorer physical functioning, higher long-term medication use (49.5%), stronger preferences for low-brightness interior color schemes (χ^2^ = 12.52), smaller and more constrained activity spaces, and lower engagement in internet use, fitness, and reading. Despite expressing the strongest willingness to modify their dwellings, this group showed low ownership of supportive facilities, indicating a demand-supply mismatch. High synchronization with family routines was observed alongside low autonomous activity participation, suggesting potential risks for autonomy and psychological wellbeing.

**Conclusion:**

This study provides cross-sectional and context-specific evidence that psychological profiles are associated with differences in residential preferences, activity participation, perceived environmental support, and facility demand among early-stage aging adults in northern China. The inclusive spatial design model should be understood as an evidence-informed design translation tool, rather than as a validated theory, causal model, or intervention protocol. Its implications are limited to preliminary guidance for aging-friendly housing assessment and housing-related public health intervention planning. Further validation through covariate-adjusted analysis, cluster-stability testing, independent samples, and longitudinal or intervention studies is required before broader theoretical, policy, or practice-level generalization.

## 1 Introduction

Population aging has made aging-friendly housing a public health priority. Existing studies have mainly examined functional decline, fall prevention, and spatial safety (1). However, many studies still treat older adults as a homogeneous group. Early-stage aging adults often retain basic independence, but their housing preferences may already differ according to mood, perceived control, safety concerns, and daily routines (2). Evidence on how these psychological differences are linked to residential preferences and home-based activities remains limited (3). This study therefore examines psychological heterogeneity as a practical factor in identifying residential demands, rather than as an abstract theoretical category.

Research on residential demands has moved from physiological compensation (4) and environmental safety (5) to broader concerns with psychological wellbeing and everyday behavior (6). This shift is consistent with the UN Decade of Healthy Aging (7), Japan's Community-based Integrated Care System (8), and China's 14th Five-Year Plan for aging services (9), all of which emphasize autonomy, social participation, mental health, and environmental support. Nevertheless, many practical policies still focus on accessibility facilities and home modification. Less attention has been given to how psychological differences among early-stage aging adults should inform inclusive residential design.

Stark et al. (5) demonstrated through randomized controlled trials that standardized flooring, circulation paths, and lighting significantly reduce fall risk. Dalvand et al. (10) confirmed in a cognitively impaired sample (*n* = 60) that modifying key spatial components and interfaces can suppress fall risk, although effects are constrained by disease progression and care dependence (11). Keall et al. (12) found in Māori households that structural safety retrofits mainly reduced fall-related injuries at the population level. Yeni and Yilmaz (13) observed in a small dementia sample that combining safety components with usage guidance reduced fall frequency. Arias-Fernández et al. (1), using a community cohort of approximately 1,800 participants, showed that clearer and more predictable neighborhood walking interfaces were associated with lower fall risk and fear of falling, highlighting the multi-scalar nature of risk prevention.

Ainsworth et al. (14), based on 20 home-modified older adults, found that environmental interventions functioned by enhancing control and activity continuity rather than solely reducing risk. Al-Homoud (15), using a sample of approximately 587 older adults, quantitatively demonstrated that home modification supports autonomy and life stability by strengthening overall control. Svensson and Stjernborg (16) analyzed older adults' mobility and social participation at the urban scale, emphasizing accessibility and environmental legibility as structural conditions for translating mobility into participation, although explanations of psychological differences remained conceptual. Ling et al. (17), in the Taiwanese context, proposed a “meaningful places” framework, arguing that spatial support must align with psychological experience and daily behavior to promote aging in place and participation. Nutley (18), using a rural sample of approximately 140 older adults, quantified associations between environmental satisfaction, quality of life, autonomy, and rhythm stability, but analysis remained at the level of overall correlations. Meng et al. (6), synthesizing evidence from more than 50 studies, identified spatial support, psychological buffering, and behavioral sustainability as shared objectives of therapeutic environments in high-density cities, yet were unable to explicate pathways of psychological type differentiation (19).

The first objective of this study is to use questionnaire surveys to examine physical function, psychological characteristics, residential conditions, and residential intentions among early-stage aging adults, thereby identifying typical residential structures and activity patterns. The second objective is to apply K-means-based questionnaire clustering to explore whether psychological heterogeneity is associated with differences in residential preferences, activity participation, and perceived environmental support. This study does not aim to establish causal pathways, independent predictive effects, or formal moderation models. Rather, it applies the established human-environment systems framework to an under-examined early-stage aging population in China. Accordingly, the contribution of this study is empirical and translational rather than theoretical. It documents psychological heterogeneity in a cross-sectional sample of early-stage aging adults, describes how such heterogeneity co-occurs with residential preferences and activity patterns, and uses these descriptive associations to generate preliminary inclusive spatial design model (ISDM)-oriented design considerations. The study does not claim that the observed profiles are universal categories or that the proposed model has been validated as a causal, predictive, or dynamic framework.

## 2 Materials and methods

### 2.1 Human-environment systems theory (HEST)

HEST originated from mid-twentieth-century interdisciplinary research at the intersection of environmental psychology and human ecology. Lawton and Nahemow (4) proposed the competence–environment press model, which posits that an individual's psychological status depends on the degree of fit between personal competence and environmental demands. When environmental press exceeds an individual's adaptive threshold, psychological distress and maladaptation may occur. This model conceptualizes psychological status as a dynamic outcome generated through the interaction between individual characteristics and environmental conditions. Subsequently, Wahl et al. (20) advanced the human–environment relational model of aging, introducing the concepts of environmental support and adaptive aging, and emphasizing the temporally dynamic relationships among psychological health, physical functioning, and the spatial environment. Within this framework, HEST is used in this study as an established theoretical lens rather than as a theory newly developed or empirically tested by the authors. It provides a conceptual vocabulary for organizing three sets of variables: psychological and functional attributes, residential environmental conditions, and behavioral patterns. Because the present study is based on cross-sectional questionnaire data and complementary home-visit evidence, it does not test dynamic adaptation, reciprocal causality, mediation, moderation, or nonlinear system effects.

**Figure 1:**
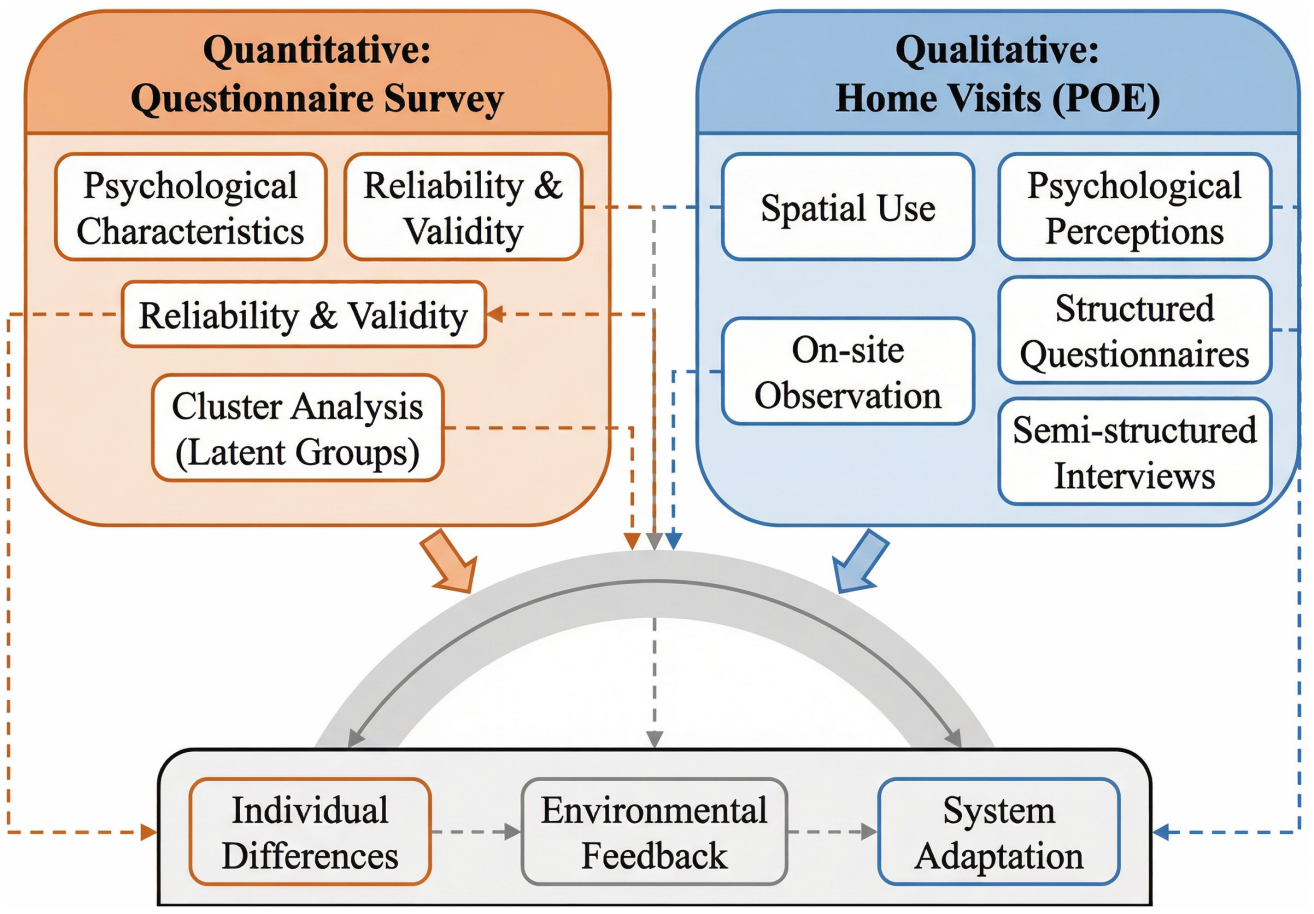
Conceptual structure of the human–environment systems framework used in this study.

HEST frames older adults' psychological status as embedded in residential settings and everyday activities. It supports clustering psychological characteristics to reveal structural differences within aging populations. The theory also emphasizes dynamic adaptation between psychological experience and spatial conditions, but in this study such links are interpreted as cross-sectional correspondences rather than causal coupling. Residential space and universal design are viewed as contexts that may relate to psychological experience and activity participation, with home visits providing contextual evidence for design translation. Finally, system-level heterogeneity suggests that different psychological profiles may respond differently to the same environment in practice. Figure 1 illustrates the conceptual relationships that guided the analysis.

From a systems science and complexity perspective, Scholz and Binder (21) further proposed that the nonlinearity and contextual dependency of HES interactions should be captured through multilevel system modeling and evidence-based integration. This approach promotes a shift from single-variable explanations toward system-coupled analysis. These studies provide the theoretical background for using HEST as an organizing lens in this study. Here, HEST is used to structure the interpretation of observed associations among psychological status, residential environmental conditions, and home-based behavior. It should not be read as an empirical test of nonlinearity, feedback loops, dynamic adaptation, or system regulation.

### 2.2 Study workflow based on questionnaire surveys and home visits

**Figure 2:**
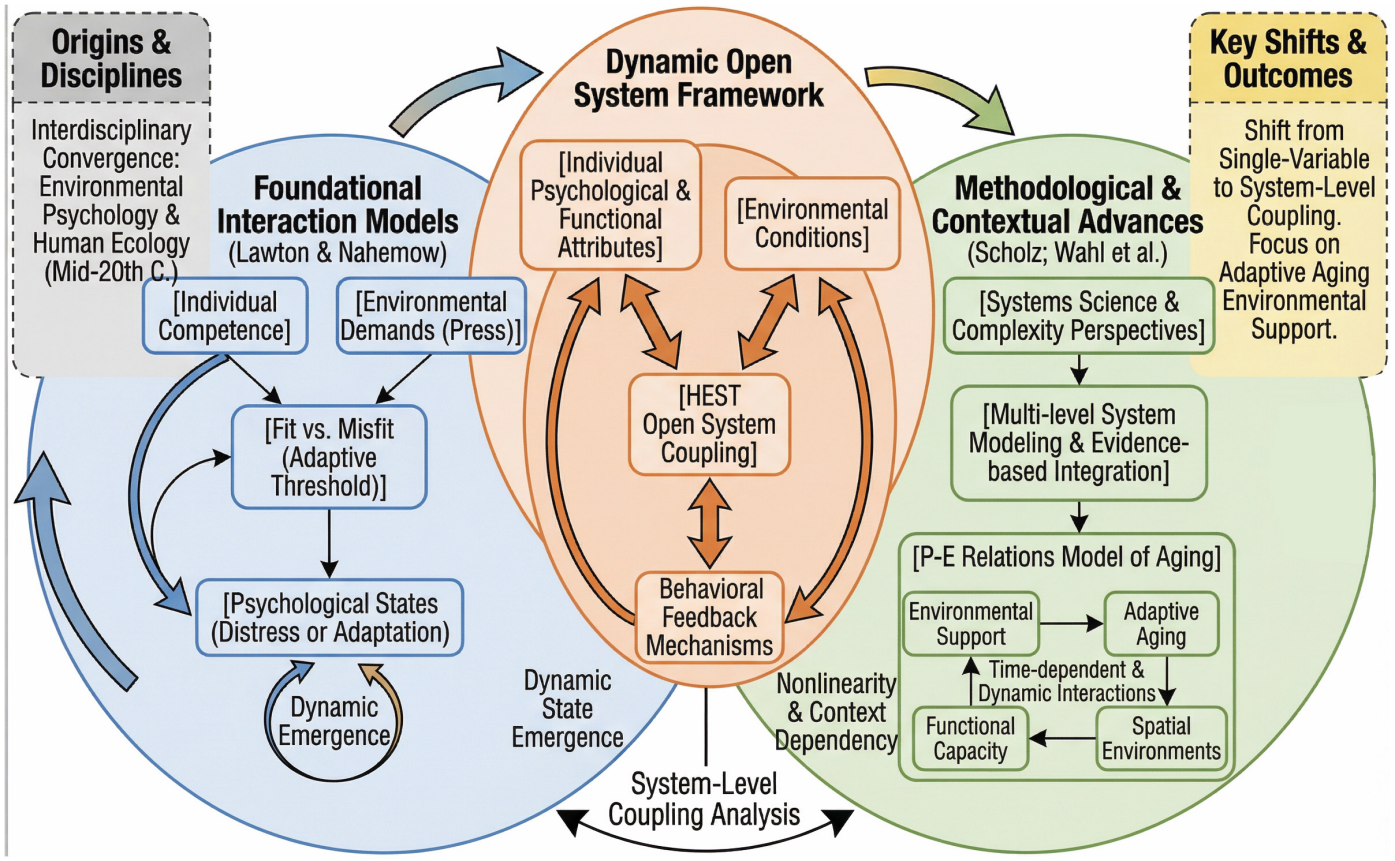
An analytical framework based on questionnaire surveys and home visits.

The research pathway consists of the following steps (Figure 2). At first, a study-specific questionnaire module was developed to measure subjective psychological experiences of older adults living in home-based aging contexts, rather than to diagnose depression, anxiety, or other clinical conditions. Item development was guided by the human–environment systems perspective, literature on aging-related psychological adaptation, case interviews, expert consultation, and a small-sample pilot test (22). Items were retained when they reflected residentially relevant experiences, including perceived privacy, safety, controllability, family interaction, daily activity continuity, emotional support, and environmental atmosphere (23). Prior to formal data analysis, internal consistency and exploratory factor structure were examined using Cronbach's α coefficients and exploratory factor analysis. These tests were used as preliminary evidence of measurement coherence and dimensional plausibility, not as proof of clinical validity. In addition, a person-centered exploratory clustering strategy was used to classify psychological characteristics among early-stage aging adults. The clustering variables were limited to the three psychological dimensions derived from the validated questionnaire structure, namely self-esteem and inferiority, loneliness and loss, and sensitivity and fear. Before clustering, all psychological variables were standardized to reduce scale-related bias. The three-cluster solution was retained as an exploratory and theory-informed typology rather than as a statistically optimal or clinically diagnostic classification. Candidate structures were assessed according to five substantive and methodological criteria: consistency with the three validated psychological dimensions, parsimony of interpretation, adequate subgroup size, distinguishability of psychological profiles, and correspondence with non-clustering residential, functional, and behavioral variables. K-means clustering was then applied to allocate individual respondents to the retained three-profile structure (24). Finally, post-occupancy evaluation (POE) was introduced as a complementary method. A subsample of questionnaire respondents was selected for in-home visits, during which spatial use patterns and psychological perceptions in real residential contexts were collected through on-site observation, structured questionnaires, and semi-structured interviews (25, 26).

By combining questionnaire-based typologies with in-home contextual evidence, this workflow supports a more situated interpretation of how psychological profiles are associated with residential demands. It does not verify a dynamic feedback process, causal pathway, or intervention effect. Therefore, the workflow supports applied design translation, but its implications remain limited to the observed sample, cross-sectional design, and home-visit context of the present study.

### 2.3 Data processing and reliability and validity testing of questionnaires

The questionnaire was developed through literature review, case interviews, expert consultation, and a small-sample pilot test (27, 28). The three psychological dimensions were named self-esteem and inferiority, loneliness and loss, and sensitivity and fear. These names should be understood as construct labels for residentially situated psychological experiences, not as established clinical scales. “Self-esteem and inferiority” refers to perceived dignity, self-evaluation, privacy-related discomfort, conflict sensitivity, and adaptation difficulty in the home. “Loneliness and loss” refers to perceived emotional support, family closeness, interpersonal connection, and the affective atmosphere of the dwelling. “Sensitivity and fear” refers to heightened risk perception, perceived environmental insecurity, worry about aging and illness, and emotional arousal in daily living contexts. The formal survey was conducted from 26 July to 8 September 2025, after institutional ethical approval and before manuscript submission. All items were scored in the same direction, with higher scores indicating stronger self-reported psychological burden or discomfort within the corresponding residentially situated construct (29). The scales were not used to classify respondents as clinically normal, depressed, anxious, or psychologically ill. No diagnostic thresholds, cut-off points, or public-health prevalence estimates were established. The scores were used only for relative comparison, exploratory clustering, and interpretation of residential demand heterogeneity.

The study was approved by the Ethics Committee of the Department of Science and Technology Management at the authors' institution (No. NCUTE-2401) and was conducted in accordance with the 1964 Declaration of Helsinki. All participants provided informed consent before participation. Data were collected mainly from Northeast China and North China, including Heilongjiang, Jilin, Liaoning, Beijing, Tianjin, and Shandong. The sampling strategy was based on targeted and convenience-based questionnaire distribution rather than probability sampling. Therefore, the sample should be understood as a regionally bounded exploratory sample of early-stage aging adults in northern China. The cultural and residential background of this sample, including relatively strong intergenerational family obligations, common co-residence or near-residence arrangements, and region-specific housing conditions, may influence how psychological characteristics are expressed in residential preferences and daily activity patterns. To reduce data-quality problems within this sampling frame, independent IP restrictions and targeted questionnaire distribution were used. A total of 575 questionnaires were returned, and 509 valid responses were retained for analysis.

SPSS was used for reliability and validity testing. Cronbach's α coefficients were first applied to assess scale reliability, followed by exploratory factor analysis to verify construct validity, ensuring that the measurement variables adequately reflected the latent structure of older adults' psychological characteristics. Variables that passed these tests were subsequently used for statistical and cluster analyses to support the examination of psychological typologies and their relationships with residential spatial demands (Tables 1, 2).

**Table 1:** Decision logic for retaining the three-cluster psychological typology in the exploratory clustering procedure.

Decision aspect	Rationale	Interpretive role in this study
Measurement basis	The three psychological dimensions were derived from the validated questionnaire structure, including self-esteem and inferiority, loneliness and loss, and sensitivity and fear.	Provides the psychological structure for subgroup classification and ensures that the clustering procedure is grounded in the measurement design rather than in arbitrary grouping.
Exploratory clustering logic	Hierarchical clustering was first used to inspect whether the standardized psychological variables formed interpretable subgroup structures. K-means clustering was then used to refine group membership.	Explains why a two-step clustering strategy was adopted and how the final typology was generated from the questionnaire data.
Cluster interpretability	The three-cluster solution produced clearly distinguishable psychological profiles: an intermediate group, a psychologically vulnerable or sub-healthy group, and a healthier group.	Supports the use of PTA, PTB, and PTC as meaningful psychological types rather than as minor statistical fluctuations.
Subgroup usability	Each cluster contained a sufficient number of cases for subsequent descriptive comparison and inferential analysis.	Avoids excessive fragmentation of the sample and allows meaningful comparison of residential preferences, activity participation, and facility demands across groups.
Analytical consistency	The retained psychological types corresponded meaningfully with differences in physical function, residential preference, activity participation, color preference, and supportive facility demand.	Links psychological classification to the human–environment systems framework and to the empirical analysis of residential demands.
Scope control	The typology is used for exploratory differentiation of residential demands rather than for clinical diagnosis or universal psychological classification.	Prevents overinterpretation of the clustering results and clarifies the boundary of the study's methodological claims.
Contribution to ISDM development	The three psychological types provide a basis for translating heterogeneous residential needs into differentiated inclusive spatial design strategies.	Connects the clustering results with the proposed inclusive spatial design model and supports the practical design-oriented contribution of the study.

**Table 2:** Clarification of clustering variables, profile description, and non-clustering external correlates.

Analytical component	Variables or evidence used	Role in the present study	How this avoids overinterpretation
Clustering variables	Three psychological dimensions: self-esteem and inferiority, loneliness and loss, and sensitivity and fear	Used to generate the exploratory psychological profiles through standardized K-means clustering	These variables define the profiles but are not treated as independent validation evidence.
Post-clustering profile description	Group differences in the psychological items reported in Table 9	Used to describe why PTA, PTB, and PTC show different relative psychological-score patterns	These differences are interpreted as profile separation and naming clarification, not as external validation.
Neutral profile labels	PTA, PTB, and PTC; descriptive terms: intermediate profile, higher-burden profile, and lower-burden profile	Used to avoid diagnostic or value-laden terms such as “normal,” “sub-healthy,” and “healthy”	The profiles are defined as relative questionnaire-score patterns within this sample rather than clinical categories.
Physical functional status	Vision, hearing, memory, limb mobility, appetite and digestion, and disease resistance	Used as non-clustering external correlates of the psychological profiles	These variables were not used to create the clusters and therefore help contextualize whether the profiles correspond to functional differences.
Medication-related care need	Long-term medication use and medication-related daily process	Used to examine whether psychological profiles are associated with health-management demands	Medication use provides external functional and care-related evidence beyond psychological questionnaire items.
Residential and environmental preferences	Bedroom color preference, residential space conditions, and perceived activity space	Used to examine whether psychological profiles correspond to differentiated environmental perception and spatial demand	These variables connect the typology to design-relevant residential conditions rather than only to psychological scale scores.
Activity participation	Internet use, fitness, reading, hobbies, shared entertainment, cooking, household chores, and daily rhythm synchronization	Used to examine whether psychological profiles correspond to different behavioral patterns at home	These variables support the interpretation that residential demand is linked to activity opportunity and autonomy.
Facility demand and facility ownership	Willingness to modify the dwelling, willingness to install barrier-free facilities, preference for grab bars, enlarged switches, safety handrails, non-slip tiles, shower seating, and actual facility ownership	Used to identify demand-supply mismatch in aging-friendly residential intervention	These variables translate the exploratory typology into practical design implications without claiming clinical diagnosis.
Methodological boundary	Absence of silhouette scores, elbow plots, cluster stability tests, cross-validation, and sensitivity analyses in the present study	Reported as a limitation and future validation agenda	This prevents the three-profile solution from being overstated as statistically optimal or universally reproducible.

To further clarify the selection and use of the cluster number, the retained three-profile structure should be understood as an exploratory and theory-informed working typology. It is not presented as a clinical diagnosis, a universal segmentation of older adults, or a machine-learning classification model. The psychological items used to generate the clusters are used only for post-clustering profile description and naming clarification (30). They are not treated as independent validation evidence. To avoid tautological interpretation, the analytical usefulness of the typology is examined through variables that were not used in the clustering procedure, including physical functional status, long-term medication use, bedroom color preference, residential conditions, activity participation, home-modification willingness, and ownership of supportive facilities.

Given the cross-sectional questionnaire design, the present study uses clustering and group comparisons to describe associations among psychological profiles, residential conditions, and home-based activities. No covariate-adjusted regression model, interaction-term model, or formal moderation analysis is estimated in this manuscript. Therefore, terms such as “effect,” “moderation,” “independent influence,” and “causal mechanism” are avoided in the interpretation of empirical results. Variables such as age, gender, marital status, income, medication use, physical function, co-residence, and housing conditions are reported descriptively and used to contextualize the observed group differences, but they are not treated as statistical controls. Future research should test these relationships using regression-based models with appropriate covariate adjustment and interaction terms.

Internal consistency was assessed using Cronbach's α coefficients. Values above 0.8 indicate good internal consistency, values between 0.7 and 0.8 are considered acceptable, subscale coefficients should not be lower than 0.7, and values below 0.6 suggest that scale revision is necessary (31). As shown in Table 3, the Cronbach's α coefficients for the three scales were 0.909, 0.874, and 0.923, respectively. These results indicate that the items within each study-specific scale were internally coherent. However, high internal consistency alone does not establish clinical validity, diagnostic accuracy, or generalizability beyond the present residential research context.

**Table 3:** Reliability analysis of the three scales.

Survey	Items	Corrected item–total correlation	Cronbach's alpha if item deleted	Cronbach's alpha
a	Feeling emotionally depressed or persistently low.	0.670	0.899	0.909
	Frequently experiencing poor sleep at night and awakening from nightmares.	0.700	0.898	
	Lacking interest in daily life and perceiving life as meaningless.	0.714	0.897	
	Often feeling lonely, isolated, and without social support.	0.690	0.898	
	Becoming more easily irritated and prone to anger.	0.604	0.903	
	Experiencing a low mood upon waking in the morning or after sleep.	0.745	0.895	
	Reluctance to accept new things and difficulty learning unfamiliar content.	0.477	0.911	
	Frequently feeling restless or inexplicably uneasy.	0.729	0.896	
	Experiencing heightened anxiety and fear regarding aging, illness, and death, and feeling distressed by others' remarks about aging.	0.709	0.897	
	Often feeling fatigued without an identifiable cause.	0.676	0.899	
b	Emotions are difficult to regulate and are easily disrupted by external stimuli.	0.697	0.850	0.874
	When facing difficulties, there is no one available to provide support.	0.724	0.845	
	I frequently experience an accelerated heartbeat.	0.648	0.858	
	Younger family members tend to avoid me and show limited emotional closeness.	0.634	0.860	
	I am unable to maintain positive interpersonal relationships with others.	0.641	0.859	
	I perceive the home environment as emotionally cold and lacking vitality.	0.718	0.846	
c	Frequently experiences an accelerated heartbeat.	0.722	0.915	0.923
	Emotional states feel difficult to regulate and are noticeably influenced by external stimuli.	0.764	0.911	
	Perceives the environment as unsafe and worries about potential risks in everyday life.	0.791	0.909	
	Often experiences poor sleep quality at night and awakens from nightmares.	0.747	0.913	
	Frequently feels unexplained worry over minor or trivial matters.	0.722	0.916	
	Regularly experiences a sense of restlessness or unease without an obvious cause.	0.826	0.905	
	Feels tense and fearful when confronting issues of aging, illness, and death, and is apprehensive about being perceived by others as old.	0.750	0.913	

Exploratory factor analysis was used to examine whether the item structure was broadly consistent with the three proposed residentially situated psychological constructs. According to Table 4, all three scales achieved KMO values of 0.959 and passed Bartlett's Test of Sphericity. Three factors were extracted, with rotated variance explanations of 22.956%, 21.236%, and 19.181%, yielding a cumulative variance explanation of 63.374%. All item factor loadings exceeded 0.5 (32). These results provide preliminary evidence that the items formed an interpretable three-factor structure in this sample. Nevertheless, exploratory factor analysis should not be interpreted as full construct validation. Confirmatory factor analysis, convergent and discriminant validity testing against established psychological instruments, and multi-sample measurement invariance tests are still required in future research.

**Table 4:** Structural validity analysis of the three scales.

Survey	Items	Factor loading coefficient		
		1	2	3
a	Feeling emotionally depressed or persistently low in mood	0.298	0.388	**0.591**
	Becoming more easily irritated and prone to losing temper	0.247	0.276	**0.699**
	Perceiving daily life as inconvenient and lacking privacy	0.240	0.364	**0.550**
	Occasionally making mistakes but refusing to acknowledge them due to concerns about self-esteem	0.179	0.172	**0.781**
	Engaging in conflicts with family members and placing strong emphasis on right and wrong	0.169	0.120	**0.791**
	Tending to interpret situations pessimistically	0.307	0.140	**0.702**
	Being reluctant to accept new things and experiencing difficulty in learning unfamiliar skills	0.136	0.225	**0.609**
b	Frequently feels lonely, isolated, and without support.	0.294	**0.646**	0.336
	Younger family members often avoid interaction and show little closeness.	0.231	**0.783**	0.163
	Shows limited interest in daily life and perceives life as lacking meaning.	0.369	**0.716**	0.198
	Experiences low mood upon waking in the morning or after sleep.	0.396	**0.652**	0.296
	Frequently feels fatigued without an apparent cause.	0.538	**0.408**	0.299
	Feels that no one provides assistance when difficulties arise.	0.378	**0.640**	0.280
	Is unable to maintain positive interpersonal relationships.	0.276	**0.654**	0.252
	Perceives the home environment as quiet and emotionally cold.	0.332	**0.670**	0.231
c	Frequently experiences an accelerated heartbeat.	**0.683**	0.328	0.232
	Emotional states are difficult to regulate and are noticeably influenced by external stimuli.	**0.699**	0.313	0.333
	Often perceives the environment as unsafe and worries about potential dangers in everyday life.	**0.733**	0.354	0.223
	Regularly experiences poor sleep at night and awakens from nightmares.	**0.683**	0.403	0.185
	Frequently feels unexplained anxiety over minor or trivial matters.	**0.776**	0.158	0.271
	Often experiences a persistent sense of restlessness without an identifiable cause.	**0.793**	0.320	0.208
	Feels pronounced tension or fear regarding aging, illness, and death, and is sensitive to others describing them as old.	**0.656**	0.399	0.251
Eigenvalue		5.050	4.672	4.220
Variance explained		22.956	21.236	19.181
Cumulative variance explained		22.956	44.192	63.374
KMO values		0.959		
Bartlett's test of sphericity		7,398.024		
Sig.		0.000		

Bold values indicate the highest factor loading for each item and identify the factor on which the item primarily loads.

## 3 Results

### 3.1 Descriptive statistics

Table 5 summarizes the respondents' basic characteristics. The age distribution is concentrated in the 55–59 group (51.5%), followed by 60–64 (22.0%) and 65–69 (13.0%), with 13.0% aged 70 and above. Females account for 55.0% of the sample and males 45.0%. Educational attainment is dominated by compulsory education (41.1%), followed by high school (33.0%) and tertiary education (23.0%). Monthly disposable income is primarily between 1,000¥ and 3,500¥ (48.1%); 25.5% report less than 1,000¥ and 20.6% report 3,500–7,000¥. Most respondents (69.9%) are no longer employed, while 23.8% continue their previous jobs. A majority (61.7%) do not require long-term medication, whereas 38.3% rely on continuous medication for disease management. The mean self-rated physical health score exceeds 3, indicating relative stability over the past 3 years, although a declining trend is evident. With increasing age, a higher proportion of older adults report reduced limb mobility, suggesting a potential association between age and functional decline (33); other physical indicators show similar patterns.

**Table 5:** Basic sample information statistics and health status.

Types	Items	Number	%
Age	55–59	262	51.5
	60–64	112	22.0
	65–69	66	13.0
	70–79	46	9.0
	≥80	23	4.5
Gender	Male	229	45.0
	Female	280	55.0
Marital status	Married	415	81.5
	Not married	94	18.5
Educational attainment	Compulsory education	209	41.1
	Upper secondary education	168	33.0
	Tertiary education	117	23.0
	Postgraduate education or above	15	2.9
Average monthly income level	< 1,000 ¥	130	25.5
	1,000 ≤ ¥ < 3,500	245	48.1
	3,500 ≤ ¥ < 7,000	105	20.6
	≥7,000 ¥	29	5.7
Employment status	No longer working	356	69.9
	Continuing in the original occupation	121	23.8
	Engaged in a new occupation	32	6.3
Medication use	Yes	195	38.3
	No	314	61.7
Physical functional status	Vision	3.39	0.95
	Hearing	3.28	0.93
	Memory	3.44	0.93
	Limb mobility	3.30	0.92
	Appetite and digestive function	3.24	0.89
	Disease resistance	3.28	1.01
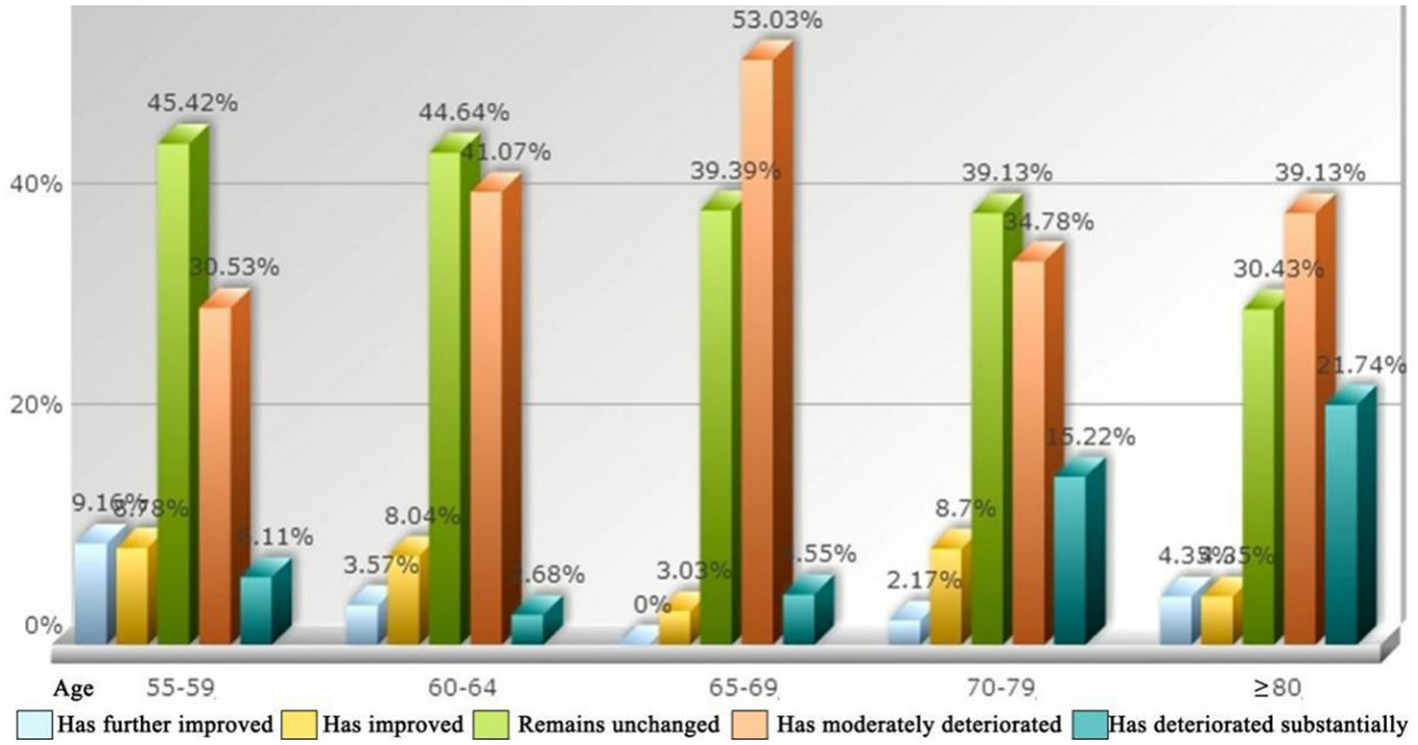 Decline in limb motor function among older adults across age groups over the past 3 years			

### 3.2 Survey on housing conditions and residential preferences

Table 6 reports residential conditions. In terms of household composition, most respondents live with their children, including co-residence with sons (47.3%) and daughters (30.1%). Approximately one-fifth live with surviving parents, and about one-fifth have grandchildren in the household, with overlapping cases. Among those with grandchildren, ages are distributed in 3-year intervals, with 26.6% under 3 years and nearly half under 6 years. Over the past 3 years, 72.3% experienced no deaths among co-residing relatives. Children's average monthly income is mainly 1,000–3,500¥ (41.7%) and 3,500–7,000¥ (30.5%).

**Table 6:** Survey on living conditions in older adults residences.

Types	Items	Number	%
Willingness of family members to live together	Other conditions	115	22.6
	Living in an adult child's household	158	31.0
	Adult children living in your household	236	46.4
	Son	241	47.3
	Daughter	153	30.1
	Daughter-in-law/son-in-law	123	24.2
	Grandchildren (including maternal grandchildren)	110	21.6
	Father/mother	106	20.8
Presence of caregiving responsibilities for grandchildren	Children under 1 year of age require full-time care.	10	9.17
	Children aged 1–3 years require continuous care.	19	17.43
	Children aged 3–6 years require ongoing care.	23	21.10
	Children aged 6 years and above require appropriate supervision.	32	29.36
	No additional care is required.	25	22.94
Occurrence of the death of a co-residing relative within the past 3 years	Yes	141	27.7
	No	368	72.3
Monthly average income level over the past year among adult children with close ties to the respondent	< 1,000 ¥	52	10.2
	1,000 ≤ ¥ < 3,500	212	41.7
	3,500 ≤ ¥ < 7,000	155	30.5
	7,000 ≤ ¥ < 15,000	60	11.8
	>15,000	26	5.1
	Unknown	4	0.8
Per capita residential floor area (m^2^)	<10 m^2^	28	5.5
	10–15 m^2^	77	15.1
	15–20 m^2^	96	18.9
	20–25 m^2^	75	14.7
	>25 m^2^	233	45.8
Availability of activity space	Very spacious	87	17.1
	Spacious	168	33.0
	Moderate	215	42.2
	Relatively narrow	36	7.1
	Extremely narrow	3	0.6
Basic bedroom conditions of older adults	Spouses sharing one independent bedroom	277	54.4
	One independent single bedroom	178	35.0
	No independent bedroom	54	10.6
	One independent single bedroom with an area exceeding 20 m^2^	25	4.9
	Spouses sharing one independent bedroom with an area smaller than 8 m^2^	46	9.0
	No independent bedroom	54	10.6
	Older adults sleeping in separate beds	258	50.7
	Per capita living area below 10 m^2^, with narrow or extremely narrow activity space	28	5.5
	Per capita living area above 25 m^2^, with very spacious activity space	189	37.1
Availability of a private toilet or bathroom	No	268	52.7
	Yes	241	47.3
Presence of difficulties in using sanitary facilities	Yes, inconvenience exists due to shared bathroom facilities	87	32.5
	Yes, inconvenience exists due to time conflicts	64	23.9
	No, no inconvenience is reported	117	43.6
Daylighting conditions of the bedroom	Predominantly natural lighting with abundant daylight exposure	313	61.5
	Natural lighting is available with occasional daylight exposure	141	27.7
	Predominantly artificial lighting with limited daylight exposure	25	4.9
	Adequate artificial lighting without daylight exposure	9	1.8
	Poor lighting conditions	21	4.1
Quality of external views from the bedroom	Open and unobstructed external view	128	25.1
	Good external view	194	38.1
	Average view quality	159	31.2
	Poor external view	23	4.5
	No external view available	5	1.0

Household size ranges from two to seven persons. Per capita living space exceeds 25 m^2^ in 45.8% of households. Only 4.9% have an independent bedroom larger than 20 m^2^; 9.0% have an independent bedroom smaller than 4 m^2^, and 10.6% have no independent bedroom. Separate sleeping arrangements are reported by 50.7% of respondents and remain close to half after excluding widowed cases. Adequate indoor activity space is reported by 37.1%, while 5.5% report limited space.

Regarding facilities, 47.3% have a private bathroom. Among those without one, 43.6% report no inconvenience and 32.5% report inconvenience. Approximately 60% report predominantly natural lighting with ample daylight in bedrooms, and nearly 30% report natural light with limited daylight. About 60% perceive bedroom views as open or good, while roughly 30% rate them as average. Overall satisfaction with bedroom lighting and views is high.

Table 7 presents residential preferences. Nearly half of respondents prefer living adjacent to their children. Monthly disposable income is associated with residential preference: those earning less than 1,000¥ tend to prefer co-residence with children, whereas higher-income respondents favor adjacent living. Among six color schemes, Scheme 6 is most preferred and Scheme 5 least preferred; warm and bright colors are favored, while low-brightness gray tones are less accepted. Most respondents prefer textured wood finishes; patterned fabrics are chosen by 47.0%, metal finishes are least preferred (13.8%), and stone and glass receive moderate preference (33.8 and 33.0%). Willingness to undertake home renovation is generally low, with mean scores of 2.57–2.77, indicating negative attitudes toward renovation investment and the installation of barrier-free facilities.

**Table 7:** Survey results on older adults residents' housing preferences.

Types	Items	Number	%
Older adults' co-residence intention (the first two items allow multiple selections)	Preference for living in close proximity to younger generations (children)	230	45.2%
	Preference for independent living or co-residence exclusively with a spouse	202	39.7%
	Preference for younger generations (children) to reside in the respondent's dwelling	107	21.0%
	Preference for residing in the dwelling of younger generations (children)	98	19.3%
Presence of a television in the bedroom	Have	324	63.7
	Do not have	185	36.3
Preference for interior finishing materials	Textured wood	444	—
	Patterned fabric	239	—
	Stone with pronounced material texture	172	—
	Glass with light-responsive properties	168	—
	Metal with a reflective finish	70	—
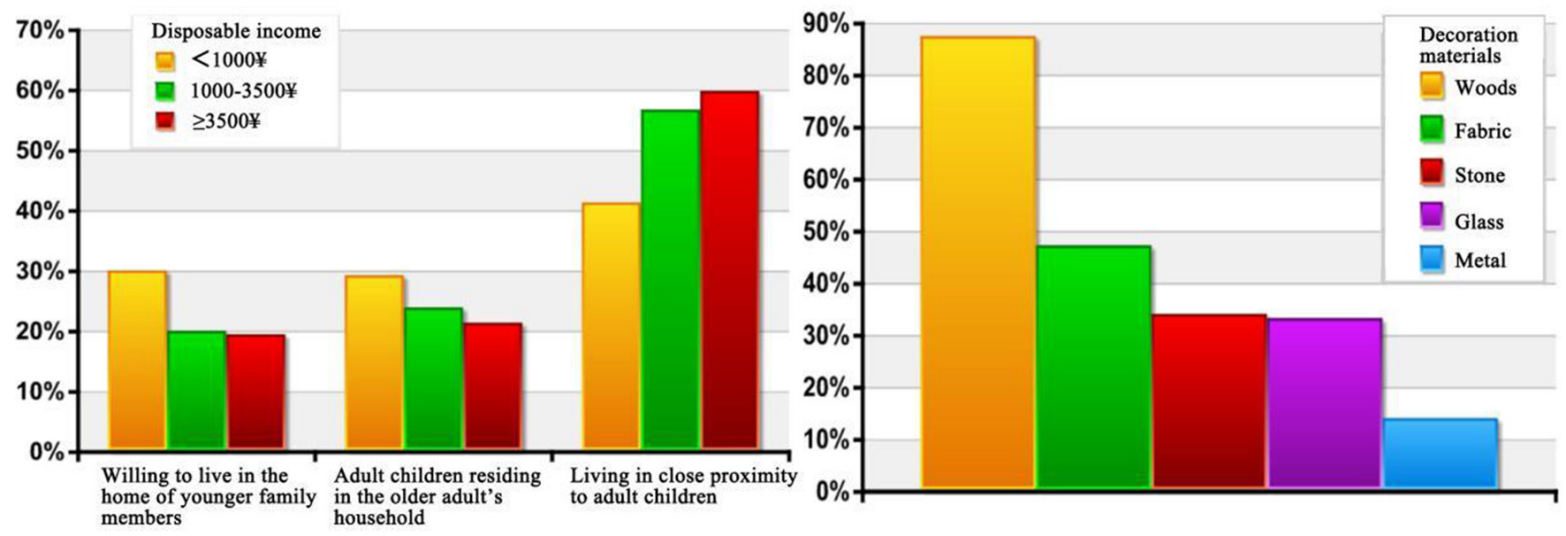			
Relationship between disposable income status and residential intention		Proportional distribution of interior finishing material selections	
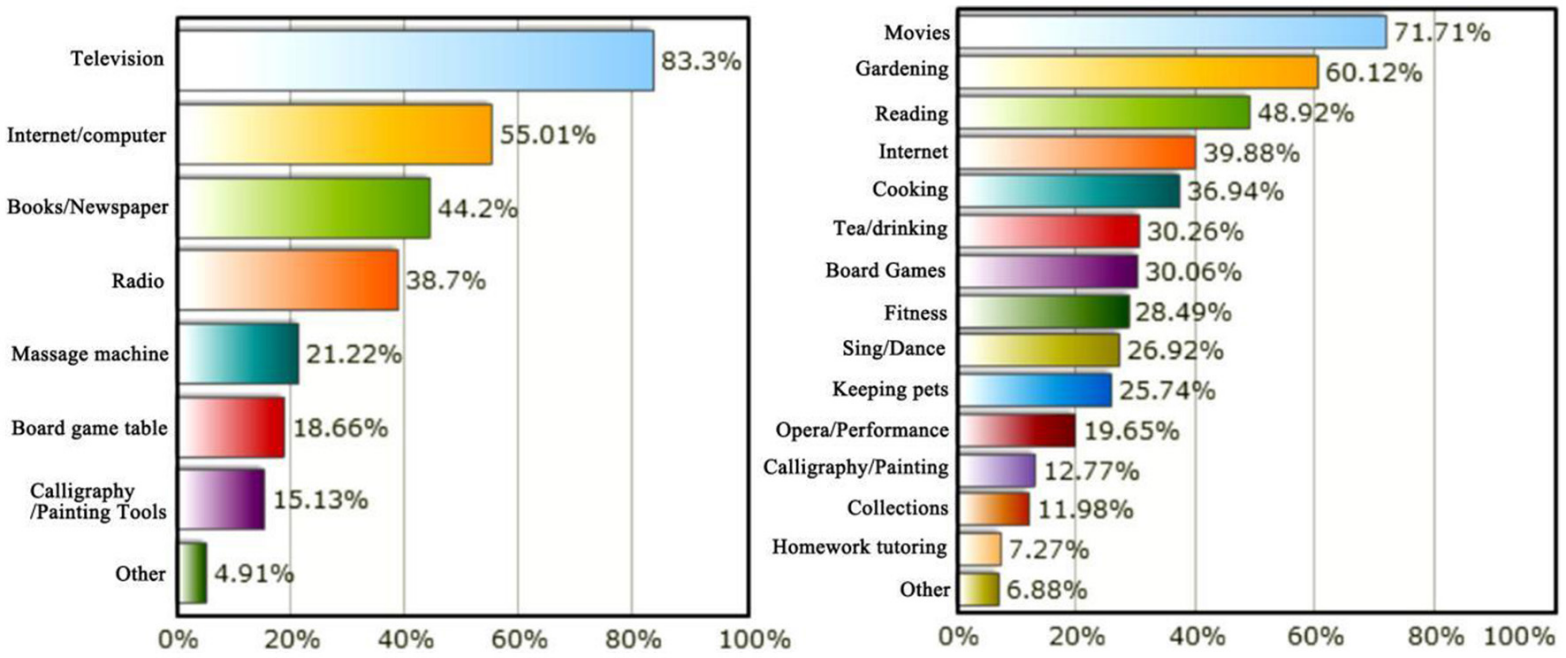			
Configuration status of recreational facilities		Home-based leisure interests of older adults	
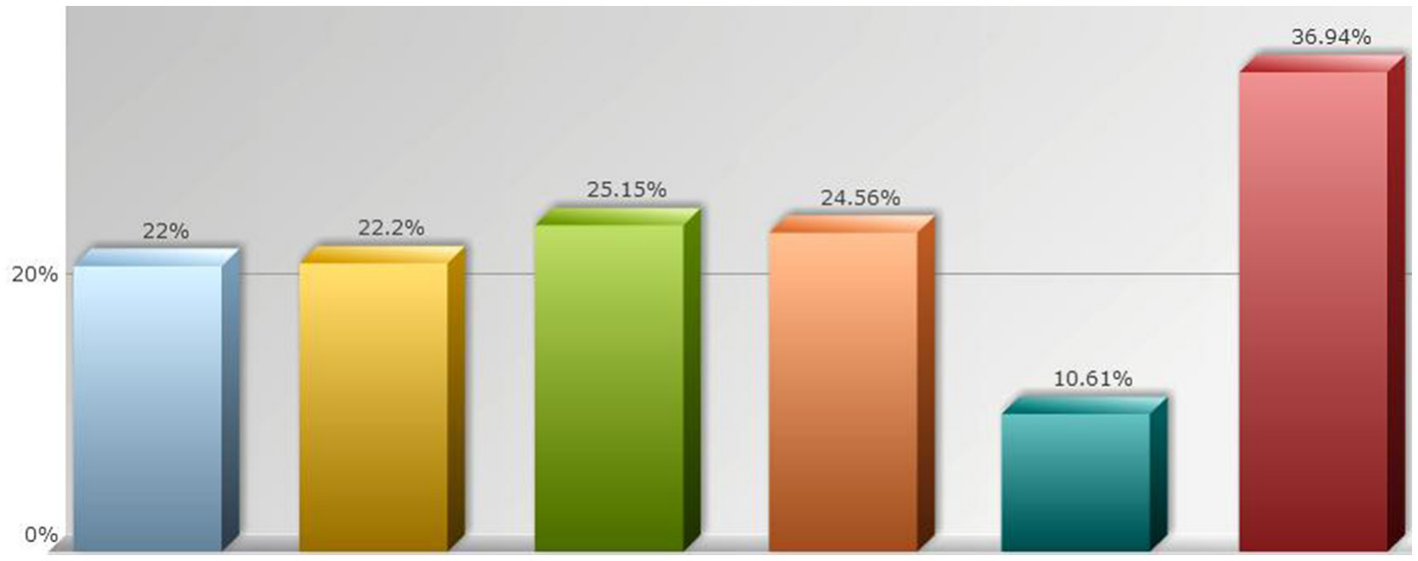 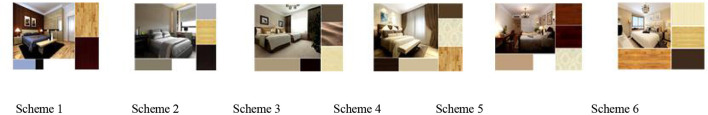			
Survey results on preferences for interior color schemes			

In terms of entertainment facilities, televisions, internet-enabled devices or computers, and books or newspapers each exceed 40% in household provision, while foot-massage devices or massage chairs are present in 21.2%. A mismatch between interests and facilities is observed: 30.06% express interest in board games or mahjong, but only 18.66% of households provide related facilities. Regarding daily habits (Table 8), overall social participation is low. Shared family meals and participation in household chores are relatively high (means of 3.78 and 3.64), whereas cooking, interest-based activities, shared entertainment, and indoor fitness show lower participation (means below 3.0). Overall, indoor entertainment and fitness activities among older adults are insufficient (34, 35).

**Table 8:** Survey on older adults' living habits.

Items	Mean	Standard deviation
Engaging in personal hobbies and interests at home.	2.85	1.03
Conversing with family members.	3.34	0.97
Participating in indoor fitness activities.	2.42	1.03
Taking part in shared recreational activities with family members.	2.73	1.02
Having meals together with family members.	3.78	1.10
Participating in household cooking activities and demonstrating culinary skills.	2.91	1.15
Performing household chores within one's physical capacity.	3.64	1.00
Demonstrating proficiency in the use of various household appliances.	3.37	1.02
Using computers, the internet, or mobile phones.	3.30	1.22
Maintaining a regular and well-structured daily routine.	3.45	1.01
Sharing similar dietary preferences with family members.	3.39	0.88
Maintaining a daily schedule that is largely consistent with that of family members.	3.28	0.95
Not experiencing frequent nocturnal awakenings.	3.03	1.02
Being able to return to sleep easily after nocturnal awakening.	3.29	1.02
Having an indoor activity range that extends beyond the bedroom.	3.37	1.09

### 3.3 Associations between psychological profiles, residential characteristics, and activity patterns

Following the decision logic described in Tables 1, 2, K-means clustering was used to derive an exploratory psychological-residential typology from the standardized psychological variables. The purpose of this section is to examine whether the resulting profiles are associated with differences in residential conditions and activity patterns, rather than to model a dynamic coupling mechanism. The three profiles are referred to as PTA, PTB, and PTC throughout the manuscript to avoid diagnostic or value-laden labeling. Based on the relative scores of the three psychological dimensions, PTA was interpreted as an intermediate psychological profile, PTB as a higher psychological-burden profile, and PTC as a lower psychological-burden profile. These labels describe relative questionnaire-score patterns within this sample and should not be read as clinical diagnoses or as judgments of personal health status. The significant differences in Table 9 indicate profile separation across the study-specific psychological experience items used to construct the clusters. These items include some affective and somatic expressions that may resemble depressive or anxiety symptoms, such as low mood, sleep disturbance, fatigue, restlessness, and worry (36). In this study, such items are interpreted only as self-reported residentially situated psychological burden, not as symptoms sufficient for clinical diagnosis. The analytical relevance of the profiles is examined in the following sections using non-clustering variables, including physical functional status, long-term medication use, residential conditions, color preference, activity participation, and facility demand. PTB was selected as the focal group because it combined higher psychological burden with poorer physical-function indicators, lower autonomous activity participation, stronger home-modification willingness, and lower ownership of supportive facilities (15).

**Table 9:** Cluster analysis table of mental health status among interviewed older adults individuals.

Survey	Items	Sample category (mean ±standard deviation)			*F*	*P*-value
		PTA	PTB	PTC		
a	Experiences persistent feelings of emotional distress and low mood.	2.72 ± 0.71	3.44 ± 0.79	1.74 ± 0.79	165.44	0.00^**^
	Displays heightened irritability and is more prone to anger.	3.05 ± 0.77	3.61 ± 0.86	1.97 ± 0.91	135.31	0.00^**^
	Perceives daily life as inconvenient and lacking in personal privacy.	2.65 ± 0.78	3.33 ± 0.80	1.78 ± 0.85	120.16	0.00^**^
	Occasionally refuses to acknowledge mistakes due to concerns about self-image.	2.95 ± 0.81	3.60 ± 0.83	2.05 ± 1.03	99.94	0.00^**^
	Engages in disputes with family members and places strong emphasis on right and wrong.	3.07 ± 0.87	3.60 ± 0.83	2.10 ± 1.05	90.58	0.00^**^
	Tends to interpret situations pessimistically.	2.99 ± 0.80	3.75 ± 0.81	2.02 ± 1.03	125.05	0.00^**^
	Shows resistance to new experiences and has difficulty acquiring new skills.	2.77 ± 0.86	3.50 ± 0.94	2.12 ± 1.09	67.04	0.00^**^
	Mean	2.89 ± 0.82	3.55 ± 0.84	1.97 ± 0.94		
b	Frequently feels lonely, isolated, and lacking emotional support.	2.63 ± 0.74	3.37 ± 0.91	1.55 ± 0.65	192.49	0.00^**^
	Younger family members often avoid contact and show limited closeness.	2.41 ± 0.78	3.35 ± 0.81	1.49 ± 0.72	181.57	0.00^**^
	Shows little interest in daily life and perceives life as lacking meaning.	2.37 ± 0.79	3.38 ± 0.91	1.29 ± 0.51	244.54	0.00^**^
	Experiences low mood upon waking in the morning or after sleep.	2.47 ± 0.73	3.36 ± 0.77	1.33 ± 0.51	286.17	0.00^**^
	Often feels fatigued without an identifiable cause.	2.88 ± 0.76	3.58 ± 0.82	1.68 ± 0.73	206.73	0.00^**^
	Perceives a lack of assistance when encountering difficulties.	2.57 ± 0.77	3.57 ± 0.73	1.55 ± 0.66	238.87	0.00^**^
	Has difficulty maintaining satisfactory interpersonal relationships.	2.40 ± 0.77	3.21 ± 0.84	1.46 ± 0.77	159.36	0.00^**^
	Feels that the home environment is quiet and emotionally cold.	2.63 ± 0.79	3.40 ± 0.81	1.53 ± 0.69	195.73	0.00^**^
	Mean	2.55 ± 0.77	3.40 ± 0.83	1.49 ± 0.65		
c	Frequently experiences an accelerated heartbeat.	2.79 ± 0.81	3.50 ± 0.86	1.65 ± 0.70	181.05	0.00^**^
	Emotional responses are difficult to regulate and are strongly influenced by external stimuli.	2.81 ± 0.72	3.58 ± 0.74	1.62 ± 0.64	258.48	0.00^**^
	Perceives the surrounding environment as unsafe and persistently worries about potential risks in daily life.	2.55 ± 0.73	3.50 ± 0.73	1.46 ± 0.63	265.31	0.00^**^
	Often experiences poor sleep quality at night and is awakened by nightmares.	2.51 ± 0.74	3.47 ± 0.74	1.53 ± 0.64	232.20	0.00^**^
	Frequently develops excessive and unexplained concerns over minor matters.	2.75 ± 0.77	3.75 ± 0.78	1.79 ± 0.86	186.57	0.00^**^
	Regularly experiences a persistent sense of restlessness without a clear cause.	2.51 ± 0.74	3.55 ± 0.72	1.50 ± 0.63	264.47	0.00^**^
	Exhibits heightened tension and fear toward aging, illness, and death, accompanied by anxiety about being perceived as old by others.	2.55 ± 0.79	3.70 ± 0.84	1.51 ± 0.61	264.14	0.00^**^
	Mean	2.31 ± 0.76	3,13 ± 0.77	1.38 ± 0.66		

^*^P < 0.05 and ^**^P < 0.01 indicate statistical significance at the 5% and 1% levels, respectively, following conventional medical-statistical reporting practice. Reference: Altman (1990), DOI: 10.1201/9780429258589.

### 3.4 The analysis of typical residential issues among older adults with the higher psychological-burden profile

The following analyses use variables that were not included in the clustering procedure to examine whether the psychological profiles correspond to differentiated residential and behavioral conditions. These non-clustering variables include physical functional status, long-term medication use, bedroom color preference, hobbies and internet use, residential space conditions, willingness to modify the dwelling, ownership of supportive facilities, and daily activity patterns. Therefore, the purpose of Sections 3.4.1–3.4.5 is not to re-demonstrate psychological differences using the same psychological items or to test a causal mechanism, but to examine whether the exploratory profiles are associated with external residential, functional, and activity-related characteristics.

#### 3.4.1 Psychological issues arising from physical conditions among different categories of older adults individuals and analysis of residential design

Based on the psychological profiles, residential issues of aging were analyzed in conjunction with non-clustering indicators of housing preference, physical function, medication use, and home-based activity. These variables were not used to generate the clusters and therefore provide external contextual evidence for the residential relevance of the typology (30). Group differences were found among psychological profiles in physical functions such as vision, hearing, and memory (Table 10). Because no multivariable covariate-adjusted model was estimated, these differences should be interpreted as unadjusted group comparisons rather than age-controlled or independently estimated effects (37). PTB had the highest mean scores across six indicators, namely vision, hearing, memory, limb mobility, appetite and digestion, and disease resistance, indicating poorer self-rated physical function, whereas PTC had the lowest scores and the best overall physical status. In terms of home-based activities, PTB older adults reported lower participation scores in computer and internet use, shared entertainment, household chores, and cooking compared with the reference groups. Chi-square analysis also showed that PTB had the highest proportion of long-term medication use. These findings indicate that the higher-burden psychological profile co-occurred with physical vulnerability, medication-related care needs, and reduced autonomous activity participation in this sample (38).

**Table 10:** Health clusters among older adults individuals with different emotional categories.

Differences in physical decline among older adults across psychological types						*F*	*P*-value
Clustering categories (mean ±standard deviation)							
**Types**	**PTA**		**PTB**		**PTC**		
Vision	3.45 ± 0.86		3.51 ± 0.97		3.17 ± 1.04	**5.59**	**0.00** ^**^
Hearing	3.38 ± 0.91		3.43 ± 0.83		3.00 ± 0.97	**9.98**	**0.00** ^**^
Memory	3.53 ± 0.85		3.67 ± 0.92		3.14 ± 0.98	**12.90**	**0.00** ^**^
Mobility of the limbs	3.37 ± 0.81		3.57 ± 0.98		3.00 ± 0.99	**13.66**	**0.00** ^**^
Appetite and digestive function	3.28 ± 0.80		3.53 ± 0.89		2.95 ± 0.97	**14.32**	**0.00** ^**^
Disease resistance	3.34 ± 0.91		3.71 ± 0.97		2.84 ± 1.04	**26.43**	**0.00** ^**^
Mean	3.39 ± 0.86		3.57 ± 0.93		3.02 ± 0.99		
**Different psychological types of older adults and the presence of chronic conditions requiring long-term medication**					**Total**	*X* ^2^	***P*** **-value**
Cluster categories (sample size, %)							
**Problem**	**Items**	**PTA**	**PTB**	**PTC**			
Whether have any chronic condition that requires long-term medication?	Yes	104 (40.8)	53 (49.5)	38 (25.9)	195 (38.3)	16.010	0.000
	No	151 (59.2)	54 (50.5)	109 (74.1)	314 (61.7)		
	Total	255 (100.0)	107 (100.0)	147 (100.0)	509 (100.0)		
Physical decline among older adults with PTB							
Clustering categories (mean ±standard deviation)							
		**Sensitivity and fear**	**Self-esteem and inferiority**	**Loneliness and loss**			
Fifty-three older adults with PTB receiving medication		3.04 ± 0.87	3.60 ± 0.93	3.67 ± 0.83			
The mean value of older adults with PTB		3.13 ± 0.77	3.55 ± 0.84	3.40 ± 0.83			
**Analysis of Physical Functional Status among Older Adults in PTB**							
**Clustering categories (mean ±standard deviation)**							
		**Sensitivity and fear**	**Self-esteem and inferiority**	**Loneliness and loss**			
Older adults with PTB whose mean scores across six indicators exceeded 3.57 (*n* = 56)		3.08 ± 0.72	3.80 ± 0.86	3.97 ± 0.92			
Overall mean scores of older adults with PTB (*n* = 107)		3.13 ± 0.77	3.55 ± 0.84	3.40 ± 0.83			
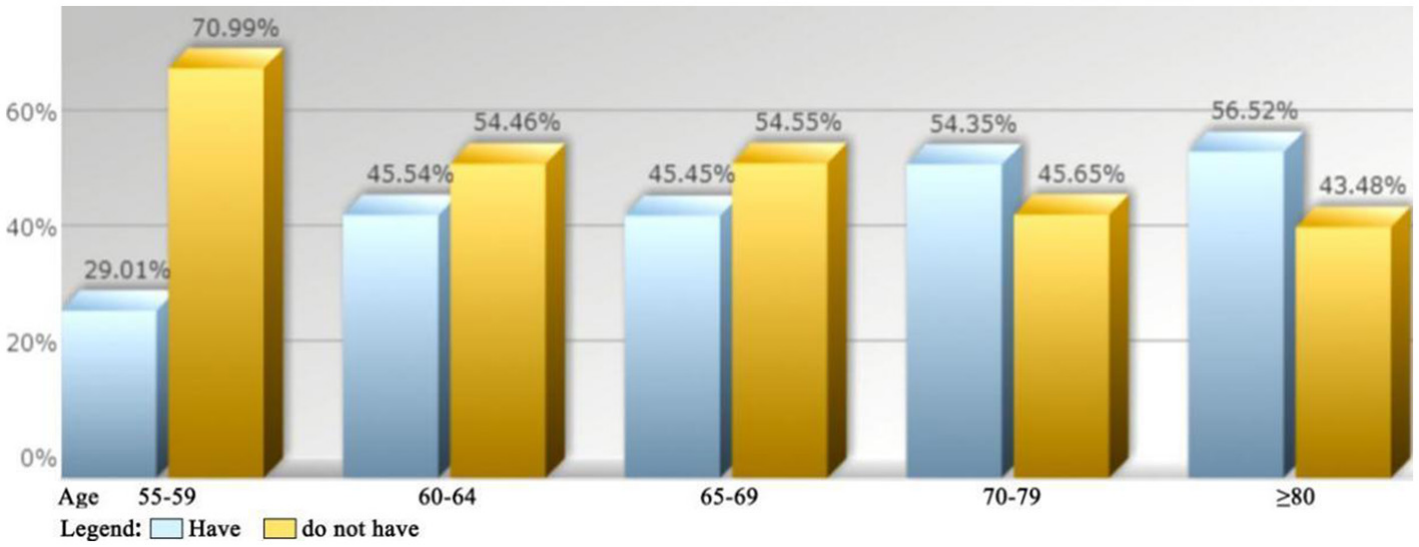 Whether the need for long-term medication increases with age							

^*^P < 0.05; ^**^P < 0.01. Bold values indicate statistically significant between-group test statistics and their corresponding P-values (*P* < 0.05). ^*^P < 0.05 and ^**^P < 0.01 indicate statistical significance at the 5% and 1% levels, respectively, following conventional medical-statistical reporting practice. Reference: Altman (1990), DOI: 10.1201/9780429258589.

Chi-square analysis revealed significant differences among psychological types regarding the need for long-term medication (χ^2^ = 16.010, *P* < 0.001). PTB showed the highest proportion (49.5%), followed by PTA (40.8%) and PTC (25.9%). Among PTB individuals requiring long-term medication, loneliness and loss scores were higher than the group mean, while differences in other psychological dimensions were not significant. Physical functional decline and long-term medication needs were more concentrated within PTB. These findings suggest that, within this sample, residential assessment for the higher-burden profile may need to pay closer attention to medication-related routines, storage convenience, and opportunities for low-threshold social and autonomous activities (39). However, because the analysis is unadjusted and cross-sectional, these design considerations should be treated as preliminary hypotheses for intervention planning rather than as evidence of direct psychological improvement.

#### 3.4.2 Psychological issues arising from color preferences among different categories of older adults individuals and their implications for residential design

According to Table 11, respondents showed relatively low preference for dark, low-lightness color schemes, with scheme 5 receiving the lowest overall acceptance. Significant differences were observed among psychological types in bedroom color preferences, particularly in the selection of scheme 5 (χ^2^ = 12.52, *P* = 0.002). PTB exhibited the highest preference proportion for scheme 5 (17.8%), exceeding that of the other psychological types, indicating relatively higher acceptance of low-lightness colors within this group.

**Table 11:** Preferences for bedroom color schemes among seniors with different emotional profiles.

Color scheme	Items	Cluster categories (number of cases, %)			Total	*X* ^2^	*P*-value
		PTA	PTB	PTC			
1	Not selected	201 (78.8)	78 (72.9)	118 (80.3)	397 (78.0)	2.166	0.339
	Selected	54 (21.2)	29 (27.1)	29 (19.7)	112 (22.0)		
	Total	255 (100.0)	107 (100.0)	147 (100.0)	509 (100.0)		
2	Not selected	197 (77.3)	79 (73.8)	120 (81.6)	396 (77.8)	2.27	0.321
	Selected	58 (22.7)	28 (26.2)	27 (18.4)	113 (22.2)		
	Total	255 (100.0)	107 (100.0)	147 (100.0)	509 (100.0)		
3	Not selected	193 (75.7)	77 (72.0)	111 (75.5)	381 (74.9)	0.603	0.74
	Selected	62 (24.3)	30 (28.0)	36 (24.5)	128 (25.1)		
	Total	255 (100.0)	107 (100.0)	147 (100.0)	509 (100.0)		
4	Not selected	188 (73.7)	90 (84.1)	106 (72.1)	384 (75.4)	5.628	0.06
	Selected	67 (26.3)	17 (15.9)	41 (27.9)	125 (24.6)		
	Total	255 (100.0)	107 (100.0)	147 (100.0)	509 (100.0)		
5	Not selected	226 (88.6)	88 (82.2)	141 (95.9)	455 (89.4)	**12.52**	**0.002**
	Selected	29 (11.4)	19 (17.8)	6 (4.1)	54 (10.6)		
	Total	255 (100.0)	107 (100.0)	147 (100.0)	509 (100.0)		
6	Not selected	170 (66.7)	70 (65.4)	81 (55.1)	321 (63.1)	5.677	0.059
	Selected	85 (33.3)	37 (34.6)	66 (44.9)	188 (36.9)		
	Total	255 (100.0)	107 (100.0)	147 (100.0)	509 (100.0)		

Bold values indicate statistically significant between-group test statistics and their corresponding P-values (*P* < 0.05).

These findings suggest that residential color design for older adults should account for differences among psychological types, with particular attention to the color preferences of PTB. Future research will further examine the effects of color lightness, saturation, and hue on psychological health across different psychological types.

#### 3.4.3 Psychological issues arising from the hobbies and interests of different categories of older adults individuals and their implications for residential design

Interests and hobbies of older adults are closely associated with residential design. Watching films and television, gardening, and reading newspapers and books were the most prevalent interests in the overall sample. As shown in Table 12, significant differences among psychological types were found for internet use, fitness activities, and reading (internet: χ^2^ = 12.24, *P*=0.002; fitness: χ^2^ = 6.474, *P* = 0.039; reading: χ^2^ = 10.55, *P* = 0.005), with PTB demonstrating markedly lower preferences. PTB also scored lower in the use of computers, the internet, and mobile phones (3.16 ± 1.13) compared with the other two groups, whereas differences in household appliance use were not significant.

**Table 12:** Psychological issues arising from the hobbies and interests of different categories of older adults individuals and their implications for residential design.

Types	Housing conditions among older adults with different psychological types						
	Items	**Types of clustering**			**Total**	*X* ^2^	***P*** **-value**
		PTA	PTB	PTC			
< 10 m^2^	Not selected	135 (52.9)	60 (56.1)	120 (81.7)	315 (61.9)	**6.55**	0.057
	Selected	35 (47.1)	47 (43.9)	27 (18.4)	194 (38.1)		
≥25 m^2^	Not selected	170 (66.7)	74 (69.2)	112 (76.2)	356 (69.9)	7.022	0.091
	Selected	85 (33.3)	33 (30.8)	35 (23.8)	153 (30.1)		
	Total	255 (100.0)	107 (100.0)	147 (100.0)	509 (100.0)		
The activity space is extremely limited	Not selected	193 (75.7)	45 (42.1)	118 (80.3)	356 (69.9)	1.421	0.892
	Selected	62 (24.3)	62 (57.9)	29 (19.7)	153 (30.1)		
The activity space is highly spacious	Not selected	201 (78.8)	76 (71.0)	94 (63.9)	321 (63.1)	1.584	0.101
	Selected	54 (21.2)	31 (29.0)	53 (36.1)	188 (36.9)		
	Total	255 (100.0)	107 (100.0)	147 (100.0)	509 (100.0)		
The bedroom area is less than 8 m^2^	Not selected	140 (54.9)	31 (28.0)	120 (81.6)	221 (43.4)	7.962	0.038
	Selected	30 (45.1)	76 (72.0)	27 (18.4)	288 (56.6)		
The bedroom area exceeds 20 m^2^	Not selected	170 (66.7)	97 (90.7)	95 (64.6)	362 (71.1)	10.019	0.052
	Selected	85 (33.3)	10 (9.3)	52 (35.4)	147 (28.9)		
	Total	255 (100.0)	107 (100.0)	147 (100.0)	509 (100.0)		
Comparative analysis of psychological status among older adults with PTB under different residential conditions							
Cluster categories (mean ±standard deviation)							
	**Excessively large residential floor area**	**Moderate residential floor area**		**Constrained residential floor area**		**Mean**	
Survey a	3.65 ± 0.96	3.04 ± 0.87		2.97 ± 0.94		3.13 ± 0.77	
Survey c	3.62 ± 0.91	3.53 ± 0.93		3.83 ± 0.97		3.55 ± 0.84	
Survey c	3.51 ± 1.01	3.43 ± 0.83		3.36 ± 0.80		3.40 ± 0.83	

Bold values indicate statistically significant between-group test statistics and their corresponding P-values (*P* < 0.05).

The results indicate that deterioration in memory, vision, and hearing reduces the capacity to learn and adopt new technologies (40). These findings highlight the need for residential design to strengthen support for internet use, fitness activities, and reading environments, thereby promoting home-based activity participation among PTB older adults (41).

#### 3.4.4 The analysis of renovation preferences and residential design for different categories of older adults individual

According to Table 13, respondents generally showed low willingness to invest in residential renovation and barrier-free facilities. Differences in renovation willingness were observed among psychological types, with PTB showing the highest mean score (3.12) and PTC the lowest. Regarding preferences for home assistive facilities, most respondents expressed neutral or positive attitudes, with particularly high demand for anti-slip flooring, bathroom seating, and window safety railings. Significant differences in facility preferences were observed across the three psychological groups. PTB demonstrated the highest preference for bathroom handrails, enlarged keyholes and switch buttons, safety railings, shower stools, and raised toilets, whereas PTC showed the lowest.

**Table 13:** The analysis of renovation preferences and intentions among older adults individuals with different emotional profiles.

Types	Different psychological types of older adults' willingness to undertake residential modifications			*F*	*P*-value
	Cluster categories (mean ±standard deviation)				
	PTA	PTB	PTC		
Willingness to invest in home renovation	2.48 ± 1.10	3.12 ± 1.23	2.48 ± 1.24	**5.80**	**0.00** ^**^
Willingness to install barrier-free facilities at home	2.76 ± 1.16	3.13 ± 1.23	2.53 ± 1.37	**7.28**	**0.00** ^**^
	**Preferences for and ownership rates of home-based assistive facilities among older adults with different psychological types**			***F***	***P*** **-value**
	**Cluster categories (mean** ±**standard deviation)**				
	PTA	PTB	PTC		
Bathroom grab bars	3.00 ± 0.98	3.35 ± 0.97	2.65 ± 1.29	12.96	0.00^**^
Enlarged keyholes and switch buttons	2.98 ± 0.93	3.43 ± 0.89	2.61 ± 1.15	21.07	0.00^**^
Safety handrails	3.35 ± 0.95	3.45 ± 0.86	2.88 ± 1.25	12.58	0.00^**^
Non-slip floor tiles	3.80 ± 0.93	3.67 ± 1.02	3.54 ± 1.28	2.67	0.07
Shower seating	3.49 ± 1.04	3.66 ± 0.96	3.05 ± 1.35	11.04	0.00^**^
Bathroom grab bars	3.07 ± 1.05	3.34 ± 0.93	2.68 ± 1.18	12.44	0.00^**^
	**Cluster categories (sample size, %)**			*X* ^2^	***P*** **-value**
Bathroom grab bars	42 (16.5)	12 (11.2)	28 (19.0)	2.421	0.192
Enlarged keyholes and switch buttons	39 (15.3)	18 (16.8)	45 (30.6)	1.567	0.098
Safety handrails	56 (22.0)	15 (14)	37 (25.2)	3.890	0.087
Non-slip floor tiles	198 (77.6)	52 (48.6)	121 (82.3)	9.451	0.057
Shower seating	22 (8.6)	6 (5.6)	12 (8.2)	1.159	0.821
Bathroom grab bars	5 (2.0)	3 (2.8)	8 (5.4)	0.677	0.959
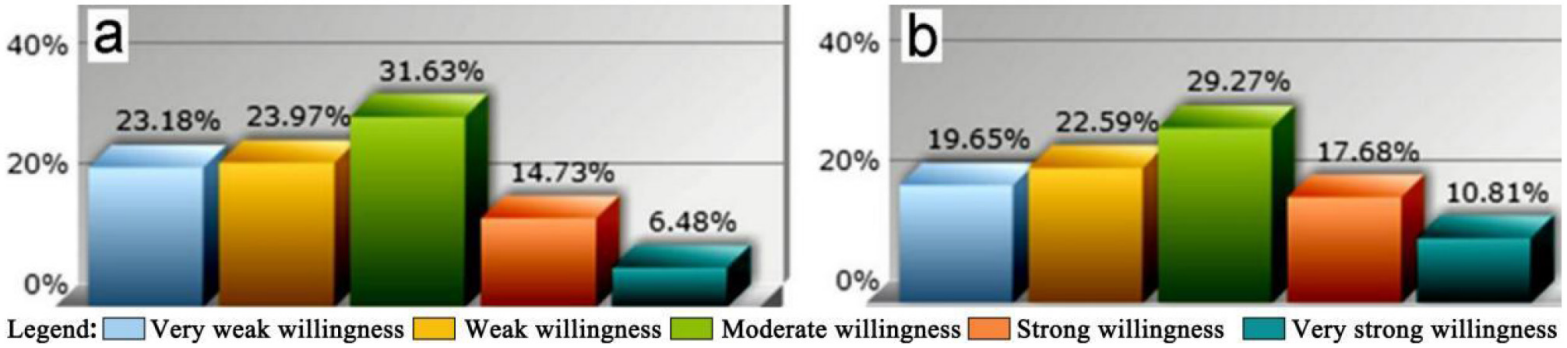					
Willingness to invest in residential modifications			Willingness to install barrier-free facilities at home		
Statistical overview of respondents' intentions toward home modification					
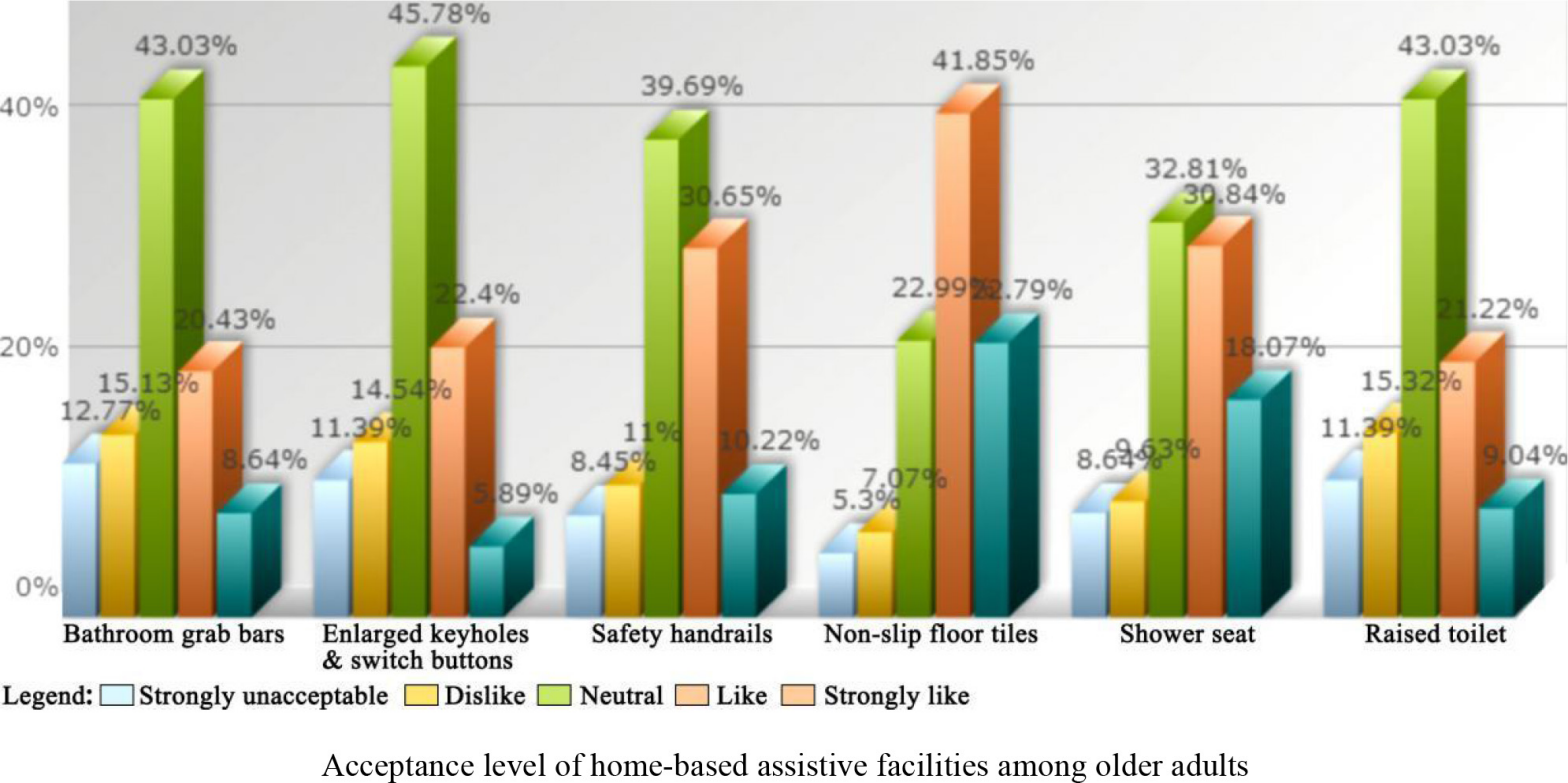					

^*^P < 0.05; ^**^P < 0.01. Bold values indicate statistically significant between-group test statistics and their corresponding P-values (*P* < 0.05). ^*^P < 0.05 and ^**^P < 0.01 indicate statistical significance at the 5% and 1% levels, respectively, following conventional medical-statistical reporting practice. Reference: Altman (1990), DOI: 10.1201/9780429258589.

Analysis of facility ownership revealed that PTB older adults had generally lower ownership rates of safety-, comfort-, and convenience-oriented facilities compared with the other groups. Within safety facilities, PTB showed lower ownership of safety railings and anti-slip tiles (safety railings: χ^2^ = 3.890, *P* = 0.087; anti-slip tiles: χ^2^ = 9.451, *P* = 0.057). These results suggest a possible demand gap in basic safety facilities among PTB participants (42). Improving such facilities may be considered in future aging-friendly housing interventions, but the present data do not demonstrate that facility installation would directly reduce sensitivity, fear, or psychological burden.

#### 3.4.5 The analysis of home activities among different categories of older adults individuals and design of senior living spaces

According to Table 14, home-based activity analysis included four dimensions: activity participation, synchronization with family members' daily life, sleep, and memory. PTB older adults exhibited the lowest levels of activity participation, with lower scores in hobbies, family communication, and indoor fitness than the other groups. In contrast, they reported higher involvement in family cooking and more frequent synchronized meals with family members. PTB showed the highest degree of daily rhythm synchronization with family members, and most did not maintain a fixed daily schedule. Interviews indicated that meal preparation aligned with family dining times or caregiving for grandchildren contributed to reduced regularity in daily routines (43).

**Table 14:** The analysis of home activities among different categories of older adults individuals and design of senior living spaces.

Types	Levels of in-home activity participation among older adults with different psychological types			*F*	*P*-value
	Cluster categories (mean ±standard deviation)				
	PTA	PTB	PTC		
Engaging in personal hobbies and interests at home	2.89 ± 0.92	2.80 ± 1.11	2.93 ± 1.14	0.98	0.38
Conversing with family members	3.26 ± 0.90	3.39 ± 0.92	3.24 ± 1.12	1.78	0.17
Participating in indoor fitness and physical exercise	2.35 ± 0.95	2.55 ± 1.11	2.45 ± 1.11	1.59	0.20
Taking part in shared recreational activities with family members	2.67 ± 0.92	2.79 ± 1.06	2.80 ± 1.14	0.90	0.41
Sharing meals with family members	3.73 ± 1.06	3.95 ± 1.04	3.60 ± 1.19	1.17	0.31
Participating in household cooking activities and demonstrating culinary skills	2.88 ± 1.10	3.07 ± 1.13	2.85 ± 1.25	1.36	0.26
Performing household chores within one's physical capacity	3.62 ± 0.99	3.64 ± 0.87	3.65 ± 1.09	0.05	0.96
Conducting indoor activities beyond the confines of the bedroom	3.35 ± 1.03	3.30 ± 0.92	3.47 ± 1.28	0.90	0.41
	**Degree of daily life synchrony between older adults with different psychological types and their co-residing family members**			***F***	***P*** **-value**
	**Cluster categories (mean** ±**standard deviation)**				
	PTA	PTB	PTC		
Engaging in shared recreational activities with family members	2.67 ± 0.92	2.79 ± 1.06	2.80 ± 1.14	0.90	0.41
Having meals together with family members	3.73 ± 1.06	3.95 ± 1.04	3.60 ± 1.19	1.17	0.31
Maintaining a stable and regular daily routine	3.43 ± 0.96	3.36 ± 0.91	3.55 ± 1.14	1.28	0.28
Sharing similar dietary preferences with family members	3.34 ± 0.81	3.41 ± 0.78	3.46 ± 1.06	0.89	0.41
Maintaining broadly aligned daily schedules with family members	3.17 ± 0.85	3.39 ± 0.81	3.28 ± 1.16	3.25	0.04^*^
	**Sleep conditions among older adults with different psychological types**			***F***	***P*** **-value**
	**Cluster categories (mean** ±**standard deviation)**				
	PTA	PTB	PTC		
No frequent nocturnal awakenings	3.02 ± 0.89	2.88 ± 1.01	2.95 ± 1.22	1.57	0.21
The ability to return to sleep easily after nighttime awakening	3.27 ± 0.94	3.21 ± 0.97	3.39 ± 1.19	1.08	0.34
Ease of sleep initiation with sustained deep sleep	3.35 ± 1.16	3.16 ± 0.91	3.35 ± 0.94	1.27	0.27
	**Memory performance among older adults across different psychological types**			***F***	***P*** **-value**
	**Cluster categories (mean** ±**standard deviation)**				
	PTA	PTB	PTC		
No episodes of forgetting essential items or steps	3.45 ± 0.91	3.69 ± 0.91	3.24 ± 1.10	1.73	0.27
No instances of intending to perform a task but forgetting it midway	3.67 ± 0.82	3.89 ± 1.16	3.28 ± 1.15	0.91	0.41
Able to readily locate items that were placed casually	3.53 ± 1.06	3.75 ± 1.04	3.40 ± 1.19	1.17	0.31
Memory performance	3.53 ± 0.85	3.67 ± 0.92	3.14 ± 0.98	12.9	0.00^**^

^*^P < 0.05; ^**^P < 0.01.

The results suggest that residential design for aging should accommodate differentiated daily schedules between older adults and co-residing family members (44). Sleep quality is closely associated with psychological status, and nocturnal awakening is a key factor affecting sleep (45). Reducing cerebral arousal during nighttime movement emerges as a critical issue (46). Memory decline was prevalent across the sample but was more pronounced in PTB. Beyond memory impairment, other factors influencing object-seeking behavior among older adults will be the focus of subsequent experimental studies (47).

## 4 Discussion

The results indicate that the surveyed older adults are predominantly in an early-stage aging condition characterized by functional independence combined with heightened environmental sensitivity. At this stage, functional redundancy has begun to contract while risks have not yet become explicit, making it a critical window in which residential interventions can achieve amplified effects (20). Spatial problems are primarily attributable to a mismatch between functional configuration and use logic rather than to insufficient floor area. Although per capita living space is relatively adequate, separate-bed sleeping arrangements and the absence of independent bedrooms are common, preventing spatial resources from being transformed into stable and controllable personal territories. This weakens perceived environmental control and intensifies demands for privacy, quietness, and order. Preferences for intergenerational co-residence reflect a rational trade-off among care provision, safety, and resource sharing under economic constraints, but such arrangements also compress individual autonomy and increase the likelihood that daily rhythms are governed by the family system (48). Residential perception is dominated by emotional attributes, with low-technology elements such as color and materials exerting a significantly stronger influence on comfort and perceived safety than spatial scale or facility complexity. Preferences for warm tones and wooden materials indicate reliance on low-threat and predictable environmental cues, whereas aversion to low-lightness gray tones suggests that most older adults do not favor emotionally withdrawn spatial environments (49). Home-based behaviors are centered on family roles and daily maintenance, with high frequencies of shared meals and household chores but limited participation in exercise, hobbies, and leisure activities. This pattern indicates insufficient spatial support for proactive and developmental behaviors and may undermine long-term physical and psychological recovery capacity (20).

Psychological type analysis shows that PTB is characterized by a high degree of overlap among emotional risk, physical functional decline, and long-term medication burden, reflecting the coexistence of physiological vulnerability and psychological risk (50). A relative preference for low-stimulation colors suggests that emotional states regulate environmental perception strategies, with individuals under high stress tending to select low-arousal spaces to achieve temporary stability (49). PTB exhibited lower activity participation but higher routine synchronization, indicating a pattern of passive synchronization within the observed household routines. This pattern may be relevant to autonomy and cognitive engagement, but longitudinal evidence is needed before interpreting it as a process of functional, behavioral, or psychological decline (16). This group shows a relatively high willingness to undertake home modifications but a low rate of safety facility ownership, revealing a structural mismatch characterized by high perceived risk and low actual provision. Overall, the observed residential problems among older adults were associated with the functional window period, spatial structural constraints, emotional regulation demands, and the passive structuring of daily rhythms. These factors were considered together to inform ISDM-oriented design interpretation, but the present study does not establish their causal ordering or independent effects. Aging-friendly home interventions may therefore prioritize small-scale spatial adjustments that reduce risk and support autonomous routines, while future research should test whether these associations remain significant after controlling for age, income, gender, marital status, health status, co-residence, and housing conditions. To make the discussion more directly connected to the empirical findings.

Cohn-Schwartz and Ayalon (51) define the late 50s to early 60s as a transition period of increasing environmental sensitivity. Our sample, dominated by respondents aged 55–59, supports this view. Lin and Chiao (48) show that housing problems are related not only to floor area but also to whether space can be used as a controllable personal domain. Consistently, our results show that adequate per capita area can coexist with shared sleeping arrangements and limited independent bedrooms. Zhao (52) further suggests that intergenerational living depends on economic conditions and preference. Our results similarly show that lower-income respondents are more likely to prefer co-residence or adjacent residence. In addition, prior studies on color, materiality, home-based activities, physical decline, and home modification (50, 53) are consistent with our findings on warm-color preference, lower activity participation, PTB's functional vulnerability, and its demand–supply mismatch in safety facilities.

In addition, the preference for living adjacent to adult children and the stronger co-residence tendency among lower-income respondents may reflect not only individual psychological needs but also family-based care expectations, filial piety norms, intergenerational responsibility, and economic risk-sharing within the Chinese aging-in-place context (54). In northern China, typical apartment-based housing, bedroom sharing, heating-season indoor life, and limited private activity areas may further intensify the association between privacy, perceived controllability, and psychological security (55). Therefore, PTA, PTB, and PTC should be interpreted as context-sensitive psychological-residential profiles rather than fixed universal categories.

Early-stage aging adults are generally not yet severely functionally impaired, making this period a preventive window for residential support rather than only a stage for disability compensation (56). Residential preference is shaped by family structure and economic conditions, as many respondents preferred living near children, while lower-income groups showed stronger co-residence tendencies. Daily life was dominated by shared meals and household chores, whereas fitness, hobbies, reading, internet use, and shared entertainment were less frequent, indicating insufficient support for autonomous activities (57). PTB participants showed poorer functional status, higher long-term medication use, lower activity participation, stronger willingness to modify homes, and lower ownership of supportive facilities, revealing compounded vulnerability and a demand–supply mismatch. Color preference also varied by psychological profile, suggesting that environmental perception is not uniform among older adults (58). PTB participants showed a co-occurrence of poorer self-rated function, higher medication use, lower activity participation, stronger home-modification willingness, and lower facility ownership, indicating a possible demand–supply mismatch within this sample. Therefore, ISDM-oriented implications should be framed as preliminary priorities for residential assessment, including privacy, lighting, circulation safety, medication-related storage, accessible activity corners, digital-use support, low-threshold exercise areas, low-cost safety facilities, and balanced support for both shared family routines and independent daily activities (59).

In addition, the design translation value of the ISDM can be further understood through recent studies on empathy-based simulation and virtual therapeutic environments, although these studies address different populations and settings. Simulating public transit experiences of individuals with mild visual impairments can cultivate empathy and improve design decision-making among students (60). Similarly, Recent study demonstrated that immersive virtual therapeutic landscapes can support anxiety-related intervention in university students, indicating the potential of spatially mediated environmental experience for emotion regulation (61). These studies do not directly validate residential interventions for early-stage aging adults; however, they provide useful methodological analogies for future ISDM development.

To engage more directly with recent empirical studies on older adults, environmental perception, and demand-oriented intervention design, Liu et al. (62) and Yang et al. (63) provide an important methodological benchmark because they examine indirect and moderated pathways linking neighborhood compactness, perceived accessibility, and mental distress among older adults. They provide a useful design-translation benchmark because they convert older adults' subjective evaluations into improvement priorities through attribute-importance and performance-based analysis. These two reports support the interpretation, methodological boundary, and design-translation logic for this study. In addition, this study also clarifies why the ISDM should be read as an evidence-informed and demand-oriented translation framework rather than as a formally tested causal or moderation model.

These results address the two research questions in a descriptive manner. First, psychological profiles were associated with differences in perceived controllability, safety, privacy, and daily rhythm in the home (15). Second, PTB participants showed stronger demand signals for privacy, safety, low-stimulation environments, and support for autonomous activities (48, 64). Because no covariate-adjusted or longitudinal model was estimated, the findings should be interpreted as evidence of association, not as proof that psychological status independently explains residential demand.

**Figure 3:**
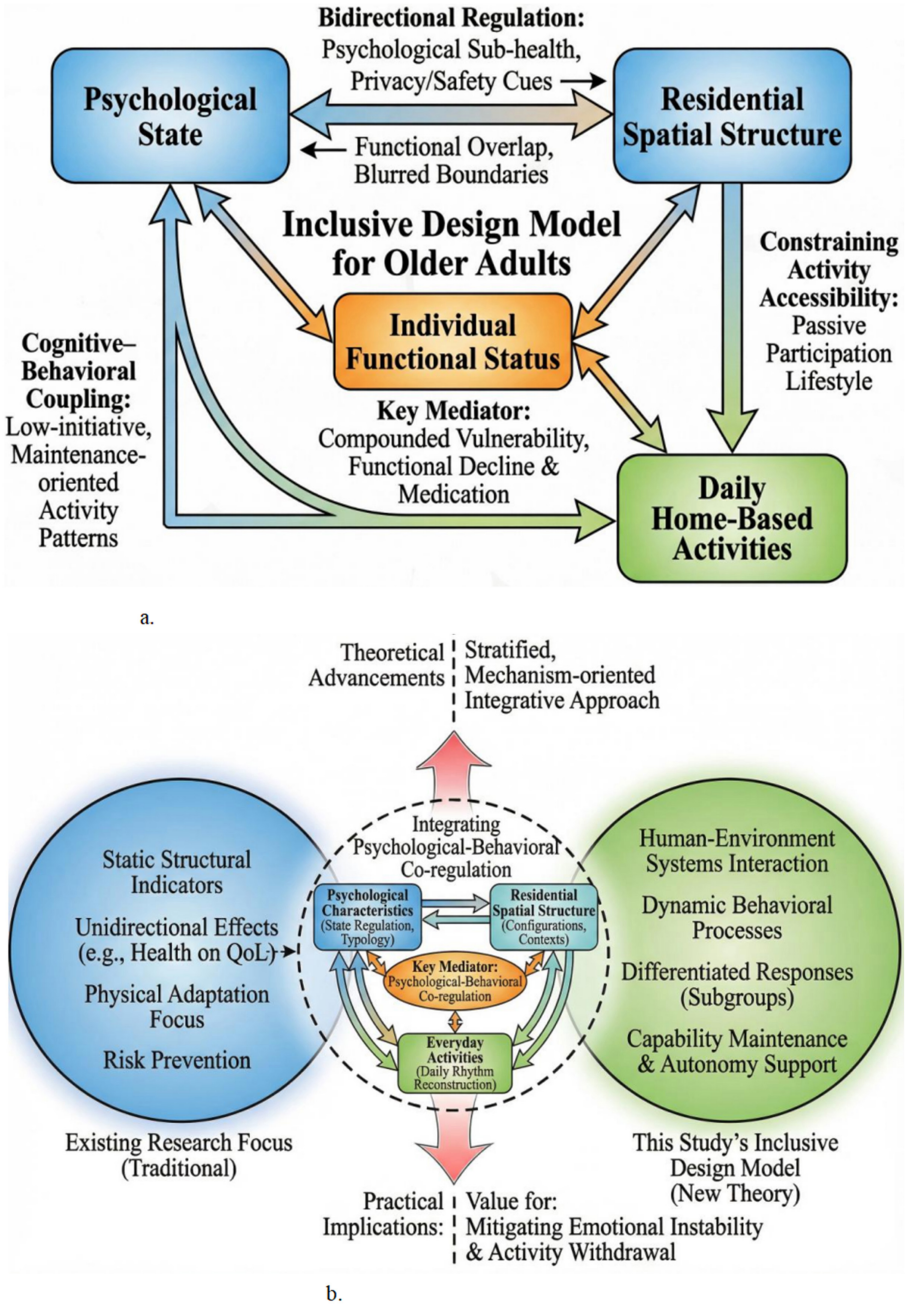
Inclusive spatial design model and academic contribution map. **(a)** The core concepts and its interaction of inclusive spatial design model. **(b)** Academic contribution map.

Based on the empirical associations identified in this study, the ISDM is presented as an application-oriented translation model rather than a new theoretical system, paradigm, or empirically tested HES mechanism. The model organizes observed relationships among psychological profiles, self-rated physical function, residential conditions, and daily activities to support preliminary differentiation in residential design assessment. Its value lies in translating psychological heterogeneity into design-relevant questions concerning privacy, controllability, activity opportunity, and facility support (65). The relationships shown in Figure 3a represent evidence-informed association pathways rather than causal mechanisms established through experimental, longitudinal, mediation, moderation, or nonlinear system modeling. Broader theoretical, policy, practice-level, or industry-wide use of ISDM requires further validation in independent samples and intervention-based studies.

Figure 3b illustrates the applied contribution of this study. Rather than proposing a new theoretical paradigm, the study uses HEST to examine an empirical problem that remains insufficiently specified in aging-friendly housing research: how psychological heterogeneity among early-stage aging adults in China is associated with residential preferences, activity participation, and perceived environmental support (66). The contribution is therefore threefold. First, the study provides sample-based evidence that early-stage aging adults are not psychologically homogeneous. Second, it shows that the PTA, PTB, and PTC profiles are linked to differentiated demands for privacy, safety, low-stimulation environments, autonomous activities, and supportive facilities. Third, it organizes these associations into the ISDM as a practical translation model for design guideline development. Thus, the model should be read as a design-oriented synthesis of empirical findings, not as a replacement for established person–environment fit or HEST theories.

The sample is concentrated on early-stage aging adults aged 55–59 with relatively high physical functioning; therefore, the conclusions are primarily applicable to community-dwelling older adults without explicit disability. Caution is required when extending the findings to older, disabled, or institution-dwelling populations, whose spatial safety needs, activity capacities, and psychological responses may differ systematically. Shen et al. (67) demonstrate that residential demands among older adults exhibit stage heterogeneity and nonlinear evolution, with structural shifts in psychological sensitivity, environmental dependence, and behavioral regulation as individuals transition from capacity maintenance to functional compensation.

One methodological limitation concerns cluster-number determination and typology validation. Although the retained three-profile solution was useful for organizing differences in residential demands, this study does not claim that three clusters represent the only statistically optimal solution. The present analysis did not report silhouette coefficients, elbow plots, cluster-stability tests, cross-validation, or sensitivity analyses across alternative cluster numbers. In addition, differences in the psychological items used to generate the clusters should not be interpreted as independent validation evidence. These psychological differences are treated only as post-clustering profile descriptions, whereas physical function, medication use, residential conditions, activity participation, color preference, home-modification willingness, and facility ownership are used as non-clustering external correlates. Future research should test the stability and reproducibility of the typology using silhouette coefficients, elbow-curve assessment, resampling-based stability tests, split-sample replication, cross-validation with independent samples, and sensitivity analyses comparing two-, three-, and four-cluster solutions.

Another limitation concerns the study-specific psychological measurement. The three scales were developed for this residential research context and should not be treated as established clinical instruments. Although internal consistency and exploratory factor analysis supported item coherence and a broadly interpretable three-factor structure, they do not prove clinical validity or diagnostic accuracy. Some items conceptually overlap because residentially situated psychological experiences often combine affective, interpersonal, and environmental perceptions (68); several also resemble depressive or anxiety-related expressions. Therefore, these items should be interpreted only as contextual indicators of self-reported psychological burden at home, not as diagnostic symptoms. Future research should compare the scales with established psychological instruments, test convergent and discriminant validity, conduct confirmatory factor analysis, and examine measurement invariance across regions, cultures, age stages, and housing types.

Further limitations concern the relationship between HEST and the empirical design, causal inference, covariate adjustment, sampling, and transferability. Although HEST includes dynamic adaptation, feedback, nonlinearity, and system coupling, this study uses these concepts only as an interpretive framework. The analysis relies on K-means clustering, descriptive statistics, chi-square tests, and group comparisons, without modeling temporal change, reciprocal causality, mediation, moderation, nonlinear system effects, or covariate-adjusted associations. Thus, “coupling” denotes observed co-occurrence and association among psychological profiles, residential conditions, and activity patterns, not a statistically tested dynamic mechanism. Although age, gender, marital status, income, medication use, physical function, co-residence, and housing conditions were collected, they were not incorporated into multivariable regression or interaction models. In addition, the targeted and convenience-based sample from Northeast and North China was not designed to represent all Chinese regions or non-Chinese contexts. Preferences for adjacent residence or co-residence may reflect filial piety, family-based care expectations, and economic interdependence (69), while northern apartment layouts, bedroom-sharing arrangements, and seasonal indoor living may amplify privacy, controllability, lighting, and activity-space concerns (70). Therefore, PTA, PTB, and PTC should be interpreted as context-sensitive psychological-residential patterns rather than universal psychological types. Future studies should use stratified multi-regional and cross-cultural samples, test cluster stability and measurement invariance, and apply regression, interaction-term testing, structural equation modeling, moderated mediation, longitudinal follow-up, repeated behavioral observation, or dynamic systems modeling to examine temporal ordering, causal pathways, and formal moderation effects.

## 5 Conclusion

The results of the study indicate that the surveyed older adults were predominantly aged 55–59 years (51.5%), with a slightly higher proportion of females (55%). Monthly income was mainly concentrated between 1,000¥ and 3,500¥ (48.1%). The mean physical function score exceeded 3, indicating a condition that is generally stable but beginning to decline. This suggests that the sample exhibits typical early-stage aging characteristics and is therefore suitable for examining aging-friendly home adaptation needs. Although 45.8% of households had a per-capita living area of at least 25 m^2^, 50.7% reported sleeping separately and 10.6% lacked an independent bedroom, indicating that overall space quantity is adequate but spatial configuration is constrained. Internal imbalance in living conditions may amplify older adults' sensitivity to privacy and psychological security. A total of 45.2% of older adults preferred living adjacent to their children, with low-income groups showing a stronger tendency toward co-residence. Bright warm color schemes and wooden materials were most preferred, whereas low-saturation dark colors were least accepted, highlighting the prominent role of emotional security in residential choice and perception. Shared family meals (mean = 3.78) and participation in household chores (mean = 3.64) were the most frequent activities, while fitness, hobbies, and shared entertainment all scored below 3. This indicates that home life among older adults is dominated by passive participation, with insufficient spatial support for active activities. Significant differences were observed among the three psychological groups, with PTB showing the highest mean scores and PTC the lowest. These results indicate a clear typological basis of psychological status among older adults, providing empirical support for subsequent differentiated spatial interventions.

PTA scored highest in indicators such as vision, memory, and disease resistance, and also had the highest proportion of long-term medication use (49.5%). Physical decline strongly overlapped with PTB, suggesting that residential design should simultaneously address physiological and psychological vulnerability. Color scheme 5 (low-saturation dark tones) had the lowest overall acceptance, yet PTB showed the highest selection rate (17.8%, χ^2^ = 12.52). This suggests that groups with negative emotional states exhibit a relative preference for low-stimulation colors, reflecting the moderating effect of emotional status on color perception. PTB demonstrated significantly lower interest in internet use, fitness, and reading, while their living spaces were more likely to feature small bedrooms and restricted activity areas. This indicates a compounded constraint among psychological status, spatial conditions, and activity participation. Overall renovation willingness among the surveyed older adults was relatively low, but PTB showed the highest willingness (mean = 3.12). Their preference for handrails and guardrails was high, whereas actual ownership was low, revealing a clear demand–supply mismatch and highlighting the intervention potential of residential safety renovations; 10 PTB exhibited the lowest level of activity participation but the highest synchronization with family routines (*P* < 0.05), alongside significantly poorer memory performance. This pattern of “passive synchronized living” may undermine autonomy and exacerbate cognitive and emotional risks.

This study examined psychological heterogeneity and residential demands among early-stage aging adults in a regionally bounded sample from northern China. Three exploratory psychological profiles were identified using study-specific, non-diagnostic questionnaire scales, and these profiles were associated with differences in residential preference, activity participation, perceived environmental support, medication-related care needs, and supportive facility ownership. The higher-burden profile showed a co-occurrence of poorer self-rated physical function, higher long-term medication use, lower autonomous activity participation, stronger modification willingness, and lower facility ownership, indicating a possible demand–supply mismatch in ordinary home-based aging settings. These findings support a cautious, evidence-informed use of ISDM for identifying design-relevant residential demands, especially privacy, controllability, low-stimulation environmental support, activity opportunities, and basic safety facilities. However, the study does not establish causal effects, independent predictors, clinical categories, or a validated theoretical model. Future studies should validate the clustering structure, use established psychological instruments, apply covariate-adjusted and longitudinal designs, and test whether ISDM-guided interventions improve residential experience and public mental health outcomes.

## Data Availability

The datasets presented in this study can be found in online repositories. The names of the repository/repositories and accession number(s) can be found below: The data used to support the findings of this study are included within the article. Data can be accessed via the following URL link: https://figshare.com/s/3a9a294121bee85c4077.
